# Low rates of bacterivory enhances phototrophy and competitive advantage for mixoplankton growing in oligotrophic waters

**DOI:** 10.1038/s41598-023-33962-x

**Published:** 2023-04-27

**Authors:** Aditee Mitra, Kevin J. Flynn

**Affiliations:** 1 School of Earth and Environmental Sciences, Cardiff University, Park Place, Cardiff, CF10 3AT Wales, UK; 2 Plymouth Marine Laboratory, Prospect Place, West Hoe, Plymouth, PL1 3DH UK

**Keywords:** Ecology, Ecology

## Abstract

With climate change, oceans are becoming increasingly nutrient limited, favouring growth of prokaryotic picoplankton at the expense of the larger protist plankton whose growth support higher trophic levels. Constitutive mixoplankton (CM), microalgal plankton with innate phototrophic capability coupled with phagotrophy, graze on these picoplankton, indirectly exploiting the excellent resource acquisition abilities of the prokaryotes. However, feeding rates can be very low (e.g., a few bacteria d^−1^). For the first time, the significance of such low consumption rates has been quantified. We find that while prokaryote-carbon (C) supply to CM grown at non-limiting light was so low that it may appear insignificant (< 10%), contributions of nitrogen (N) and phosphorus (P) from ingestions of 1–12 prokaryotes d^−1^ were significant. Under limiting light, contributions of ingested C increased, also raising the contributions of N and P. The order of nutritional importance for CM growth from predation was P > N > C. Further, provision of N through internal recycling of ingested prey-N stimulates C-fixation through photosynthesis. Importantly, coupled photo-phago-mixoplanktonic activity improved CM resource affinities for both inorganic and prey-bound nutrients, enhancing the nutritional status and competitiveness of mixoplankton. With warming oceans, with increased prokaryote abundance, we expect CM to exhibit more phagotrophy.

## Introduction

Planktonic primary production is a cornerstone process in marine ecology, supporting life in the oceans. Climate change is seeing an expansion of oligotrophic zones in the oceans^1,2^. Prokaryotic picophytoplankton and bacteria are the most numerous self-replicating marine microbes^3,4^, being resilient to multi-stressors and well adapted to life in oligotrophic conditions, resulting in an ability to out-compete the relatively larger protists for limiting dissolved resources^5^. There is evidence that the spread of oligotrophic zones will shift the plankton community structure away from protists and towards an increased abundance of these picoplankton^6^. These expectations are couched in the context of the traditional phytoplankton-zooplankton food web paradigm. However, recently this paradigm has been brought into question, with important implications for how the success of plankton communities under climate change may actually play out; it transpires that many protist ‘phytoplankton’ are mixotrophs that can eat prokaryotes.

Mixotrophy, the coupling of phototrophy and heterotrophy, has long been recognised as an important nutritional strategy for various plankton, including being associated with harmful algal bloom events^7^. It is likely that all microbial phototrophs are mixotrophic, using a form of heterotrophy called osmotrophy (the exploitation of dissolved organic nutrients). The last decade, however, has seen an upsurge in interest in plankton engaging in photosynthesis and in predation by phagotrophy^8^. As these photo-phagotrophic organisms comprise an important distinct functional group within microbial planktonic communities^9^, the term ‘mixoplankton’ has been proposed to differentiate phototrophic organisms capable of phagotrophy, from those phototrophs (i.e., the ‘phytoplankton’) that are mixotrophic via only osmotrophy^10,11^. The mixoplankton paradigm^10,12^ thus reimages the base of the marine food web raising questions over the significance of the role of phagotrophy in supporting phototrophy, especially under climate change.

**Figure 1:**
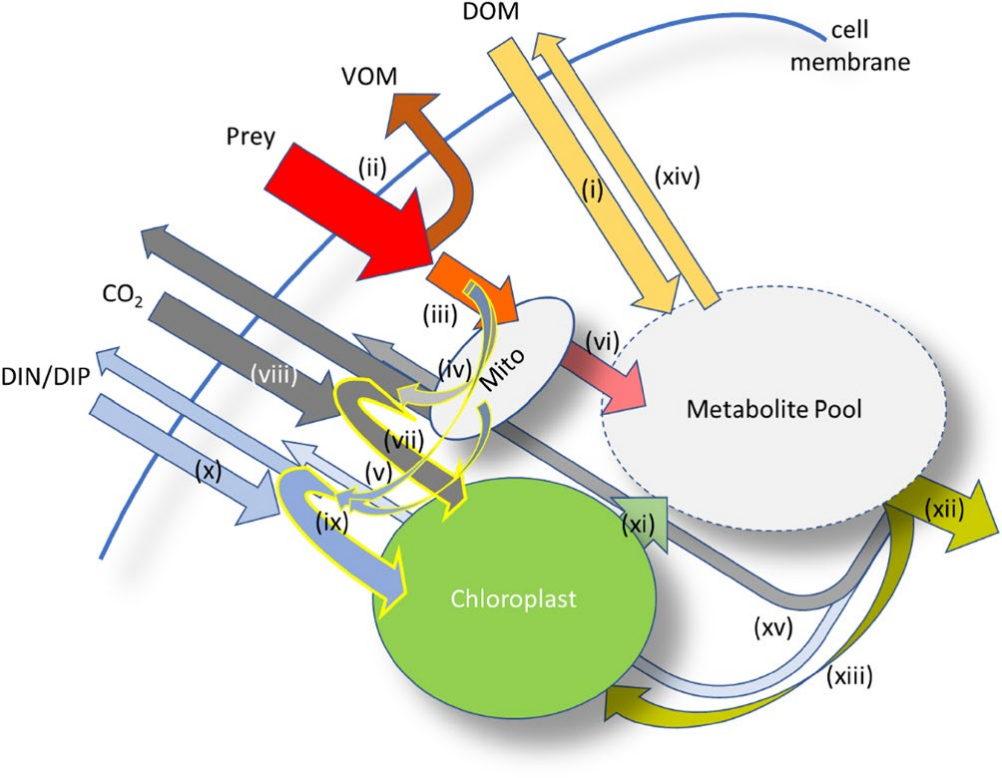
Schematic showing the coupling of physiological processes within a mixoplankton cell, supporting the synergism between phototrophy and phagotrophy. (i) Dissolved Organic Matter (DOM; sugars, amino acids etc.) is taken up and enters the metabolite pool; this action supports osmotrophy. (ii) Prey are engulfed, and a fraction (ca. 20–40%) egested as voided organic matter (VOM) during digestion. (iii) The retained fraction is broken down and a further fraction (ca. 30%) is lost through specific dynamic action (SDA) as (iv) CO_2_ and as (v) dissolved inorganics (DIN, nitrogen as ammonium; DIP, phosphorus as phosphate). This activity (iii-v) is associated with the mitochondria (Mito) and other sub-cellular compartments. (vi) The resultant remaining material enters the metabolite pool. (vii) The CO_2_ lost through SDA contributes to satisfying CO_2_ demands for photosynthesis in chloroplasts (yellow edged arrow). (viii) Any additional CO_2_ demand is brought in from outside of the cell. (ix) DIN/DIP lost through SDA contribute to DIN & DIP demands for phototrophy (yellow edged arrows). (x) Any additional demand for DIN and/or DIP over that supplied by recycling is brought in from outside. (xi) Products from phototrophy contribute to the metabolite pool. The total metabolite pool supports (xii) biomass growth including (xiii) synthesis of chloroplasts. (xiv) Excess metabolites are leaked out of the cell. (xv) Additional losses include loss of CO_2_ through respiration, with allied regeneration of DIN (as ammonium) and DIP (as phosphate) to maintain cellular stoichiometric balance. The metabolite pool equates to ^M^C in the model, as shown in Fig. S1.

Mixoplankton occur in low nutrient oligotrophic and in mature (e.g., low inorganic-nutrient summer temperate) systems^13–16^. Various exemplar members of the primary producing ‘phytoplankton’ community are now recognised as constitutive mixoplankton^9^ (CM), due to their innate phototrophic abilities coupled with an ability to ingest prokaryote prey; examples include *Emiliania huxleyi*^17^, *Phaeocystis globosa*^18^, *Heterosigma akashiwo*^19^, *Prymnesium polylepis*^19^, and *Teleaulax amphioxeia*^20^. Mixoplanktonic activity in such organisms is a fully integrated physiological process (Fig. 1), not just a top-up mechanism employed under unfavourable conditions^21–23^. While evidence shows that consumption of prokaryotes by nano-sized CM occurs in oligotrophic waters^24,25^, various studies suggest that the ingestion rates may be very low – of only a few bacterial prey per day^17,18,20^. The question then arises as to how such low ingestion rates could be of physiological significance.

Here we consider the significance of low ingestion rates of prokaryote picoplankton prey for constitutive mixoplankton. We explore the topic using a simple stoichiometric and allometric approach to obtain a broad appreciation of the potential, and then via a detailed physiological simulation model to study the important subtleties of the synergism in the interactions between phototrophy and phagotrophy (Fig. 1). We hypothesize that low bacterivory rates act as more than a survival strategy for the CM. Our analyses show that low rates of consumption of prokaryote prey can significantly support the growth of mixoplankton and also, through the physiological synergies between phototrophy and phagotrophy that also enhances affinities for both inorganic nutrients and prey. We discuss the implications of our results for field work, noting that ingestion rates of ecological significance may be so low as to go undetected in routine assays.

## Results

### Stoichiometric & allometric analysis

**Figure 2:**
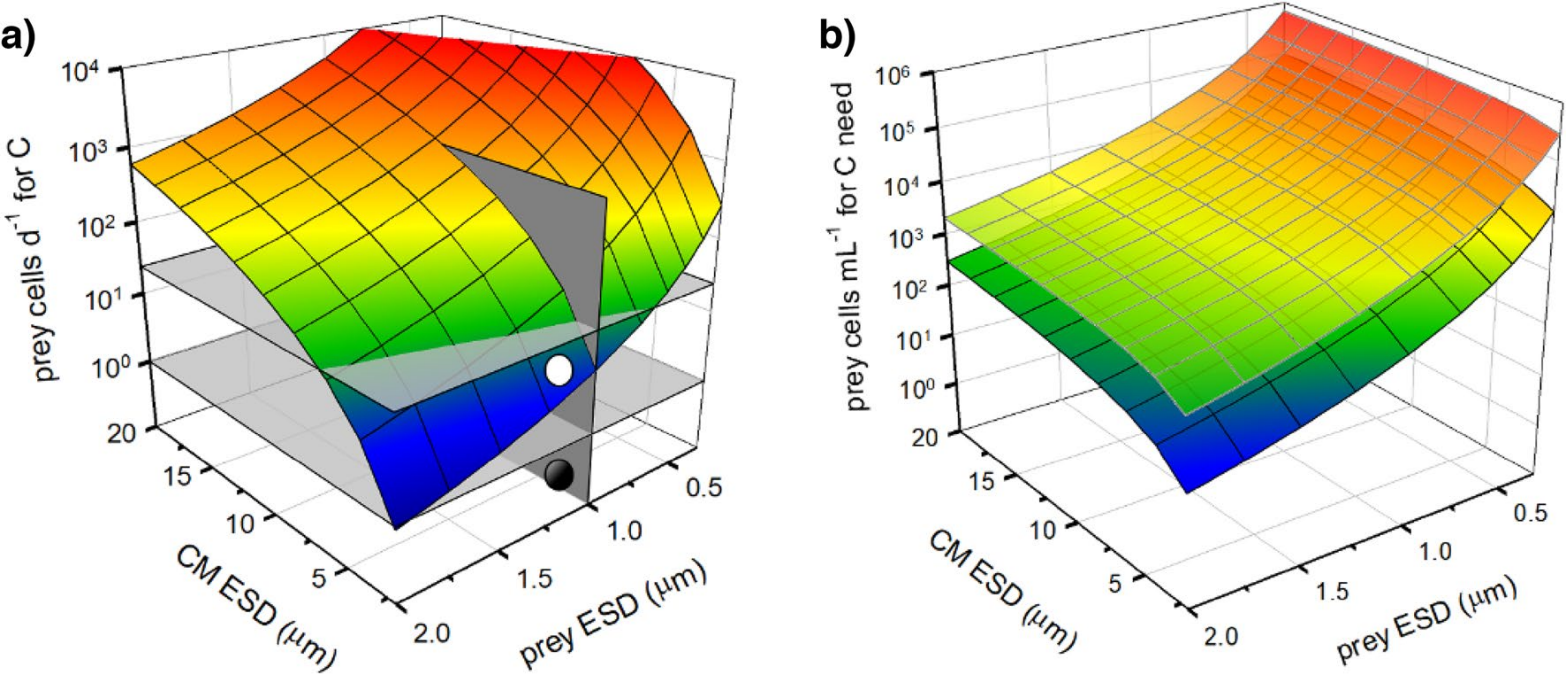
Assimilation rates of prokaryote picophytoplankton prey cells per mixoplankton cell required to satisfy C structural needs for the replication of mixoplankton cells of different sizes. In Panel (**a**), the horizontal plane at 10^0^ is for an ingestion rate of 1 prey d^−1^; a mesh in the zone below this plane (dark circle) indicates that fewer than one ingestion event per day would satisfy the demand. The horizontal plane at 10^1.38^ indicates an ingestion rate of 24 prey d^−1^ (i.e., an average of 1 prey h^−1^); the mesh in the zone between this plane and the lower plane (white circle) indicates that ingestion events between 1 and 24 prey d^−1^ would satisfy demand. Panel (**b**) shows the prey abundance required to support the grazing rates in panel (**a**) assuming ingestion of all encountered prey under conditions of no turbulence (upper mesh), and with turbulence (1e-3 m s^−1^; lower mesh). ESD, equivalent spherical diameter. See Table S1 for stoichiometric values.

For acquisition of all elements (C,N,P) through phagotrophy there was a curvilinear relationship between prey size and the prey ingestion rate per day needed to satisfy the structural demands for that element at a growth rate of 0.693 d^−1^ (i.e., a doubling per day); this was coupled with another curvilinear relationship with mixoplankton cell size (Figs. 2, S4, S5). The lower left corner of Fig. 2a, where prey and predator are both of ESD 2 µm, shows the expected results that consumption of 1 prey per day would supply all the required structural C for a CM doubling per day; such an event is only likely in reality in instances where ingestion of prey-digestate occurs via a feeding tube^26^. The corresponding prey abundance field (Fig. 2b, with or without turbulence which enhances prey encounter rates), assuming 100% efficiency in capture upon encounter, shows that prey abundances around 10^3^–10^4^ mL^−1^ are required to support the events shown in Fig. 2a. The analogous plots for N and P from cyanobacterial picophytoplankton (Fig. S4), and of C, N, and P from bacteria (Fig. S5) all show trends similar to Fig. 2 but with different levels of nutritional support for a given ingestion rate.

**Figure 3:**
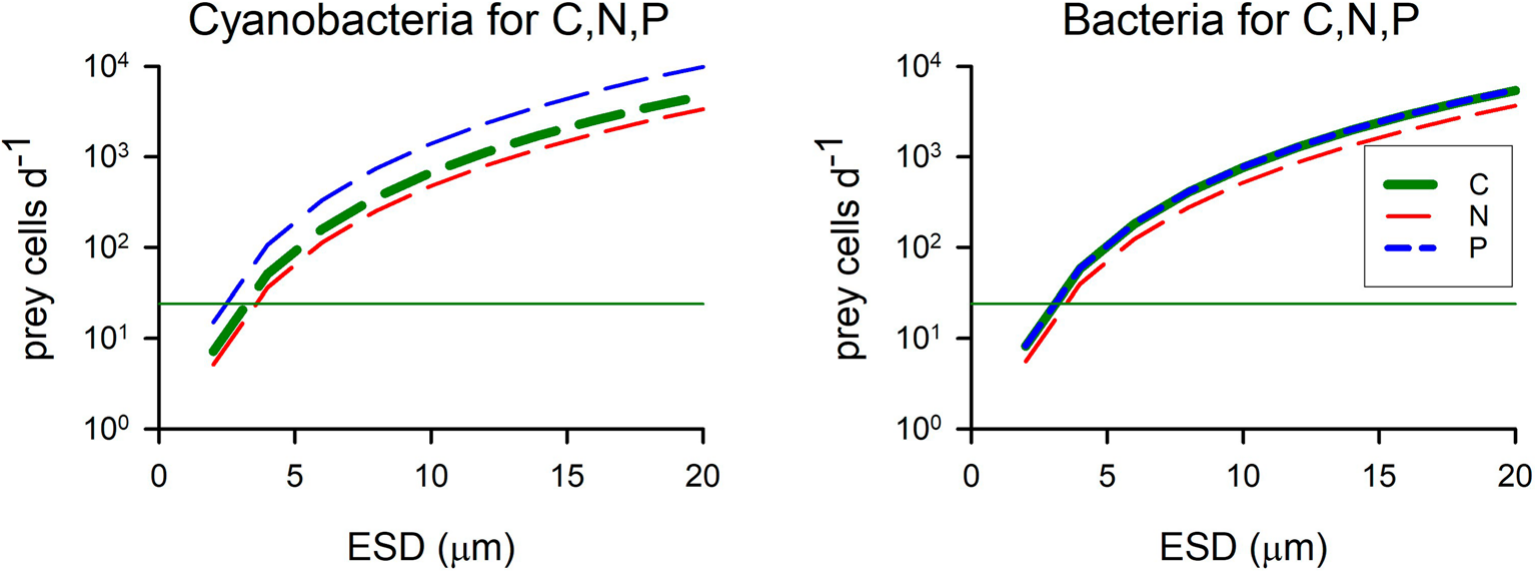
Ingestion rates of prey cells per mixoplankton cell required to satisfy all C,N,P structural needs for the replication of mixoplankton cells of different sizes. Prey were all assumed to be 1 µm ESD, as either heterotrophic bacteria, or as picophytoplanktonic cyanobacteria. The thin horizontal line, at 10^1.38^, indicates an ingestion rate of 1 prey h^−1^. See also Fig. 2, and Figs. S4, S5.

Data for bacterial or cyanobacterial prey of 1 µm ESD, as shown in Fig. 2 and Figs. S4 and S5, are co-plotted in Fig. 3, showing required assimilation rates of the different sized predator CM for all element types. Cyanobacteria are more C and N dense, but contain less P than similar-sized bacteria, the ratio of the assimilation rates as cyanobacteria:bacteria are 0.87 for C, 0.92 for N and 1.8 for P (Fig. 3). From this simple analysis, a 5 µm ESD CM would appear to need to assimilate around 100 prokaryote prey per day (ca. 1 every 15 min) to satisfy its needs. However, to support a critical minimum growth rate to compensate for loss through mixing out of the photic zone (ca. 0.03 d^−1^)^27^, ingestion rates of only ca. 2 prey d^−1^ would suffice.

### Analyses of the simulations

**Figure 4:**
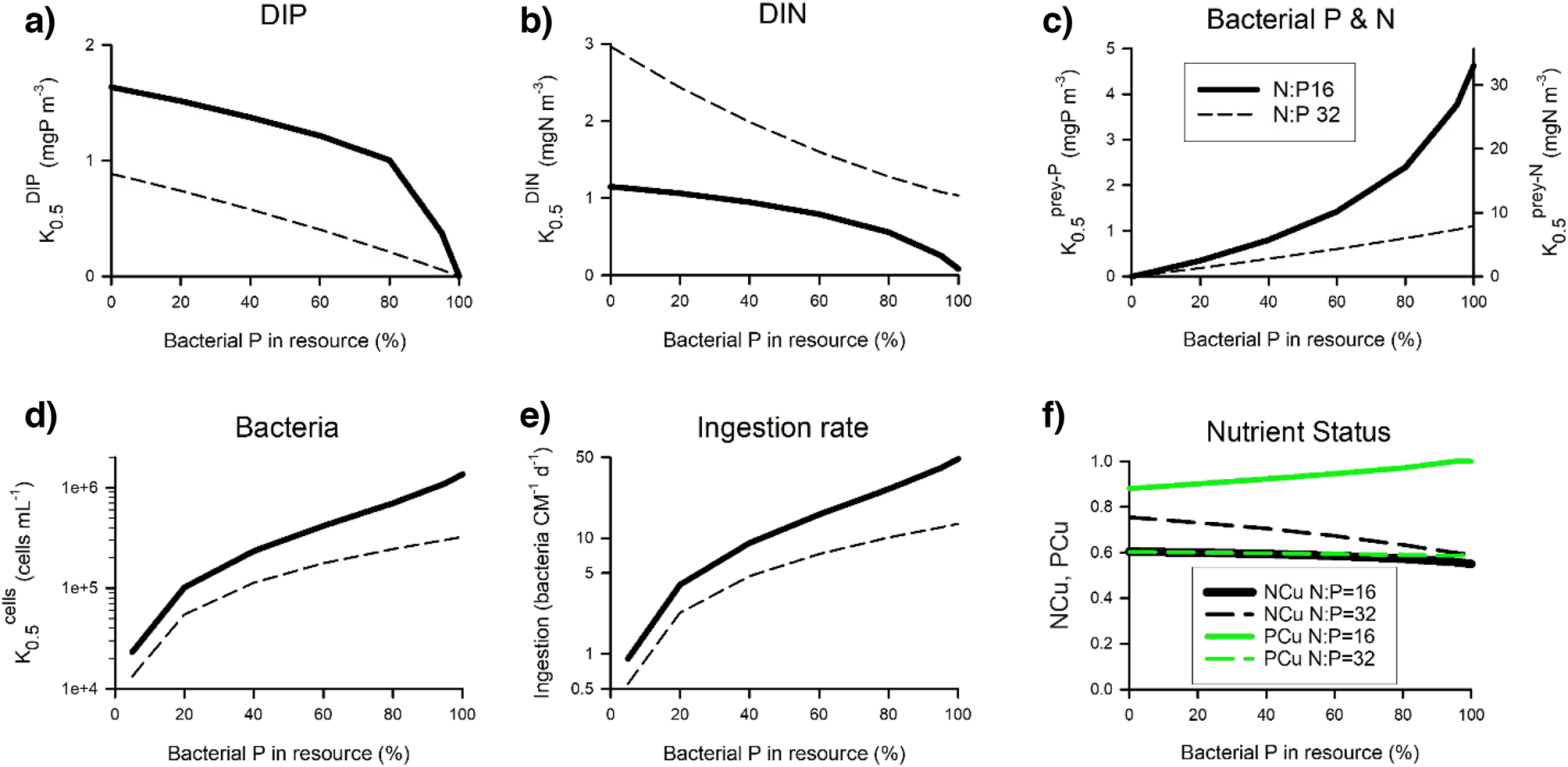
Relationships between the proportion of nutrients provided via phagotrophy and emergent half saturation values for resources supplied as inorganics (DIP, DIN) and/or as bacteria. These are outputs from the chemostat simulations run at a dilution rate to enable the constitutive mixoplankton (CM) to grow at half the maximum growth rate (i.e., as required to derive values of K_0.5_). Data are shown for a CM of 5 µm ESD, growing with a total resource abundance of 70 mgN m^−3^, associated with a total mole N:P of 16 or 32, with the indicated % of the P allocated as bacterial P (x-axis). K_0.5_ values for DIP, DIN and bacteria are shown in panels (**a**), (**b**) and (**c**) respectively; the bacterial C:N:P was fixed, hence the same plot lines are read for K_0.5_^prey-P^ and K_0.5_^prey-N^ on different y-axes in (**c**). Panel (**d**) shows K_0.5_ for bacteria cell numbers, with the ingestion rates in panel (**e**). Panel (**f**) shows the emergent N and P nutrient status of the mixoplankton, where 1 is replete and 0 is starved; only at resource N:P = 32 was P-status (PCu) lower than that for N (NCu). See Table S1 for stoichiometric values and Fig. S6 for the proportions of C, N and P acquired from feeding.

The simulation model describes the physiological synergies of photo-phago-mixotrophy (Fig. 1), enabling a more nuanced appreciation of the potential of phagotrophy upon prokaryote prey. Simulations of mixoplankton growth under different availabilities of inorganic versus bacterial-bound nutrients allow the calculation of resource affinities (K_0.5_ values); these change as the balance of phototrophy and phagotrophy varies with the availability of inorganic nutrients or prey (Fig. 4). K_0.5_ for inorganic resources (Fig. 4a, b) versus prey resources (Fig. 4c) were in the same range for P (K_0.5_^prey-P^ ≈ K_0.5_^DIP^), but different for N (K_0.5_^prey-N^ >  > K_0.5_^DIN^); feeding for P is thus more effective than for N, and the latter would be more important in waters with high prey abundance. Ingestion rates when resources for P were split equally between DIP and prey-P were ca.1 bacterium consumed every few hours, with K_0.5_^cells^ around 2 × 10^5^ bacteria mL^−1^ (Fig. 4d). Differences in ingestion rates (Fig. 4e) reflect the interplay in photo-phago-physiology. Values for K_0.5_^DIP^ (Fig. 4a), K_0.5_^DIN^ (Fig. 4b), K_0.5_^prey-P^ & K_0.5_^prey-N^ (Fig. 4c), and, K_0.5_^cells^ for prey (Fig. 4d) differed under resource regimes of N:P = 16 versus N:P = 32. P was more limiting than was N with N:P = 32, but this limitation was not so pronounced with higher allocations of resource P within bacteria (Fig. 4f). The proportion of P from bacteria that contributed to CM growth closely tracked the proportion supplied under both N:P resource regimes (Fig. S6). The contribution of prey biomass to mixoplankton needs was much lower for N than for P (< 50% of the P supply), and lower again in terms of C (< 20% of the P supply); these differences were greater for N:P = 32 (Fig. S6).

**Figure 5:**
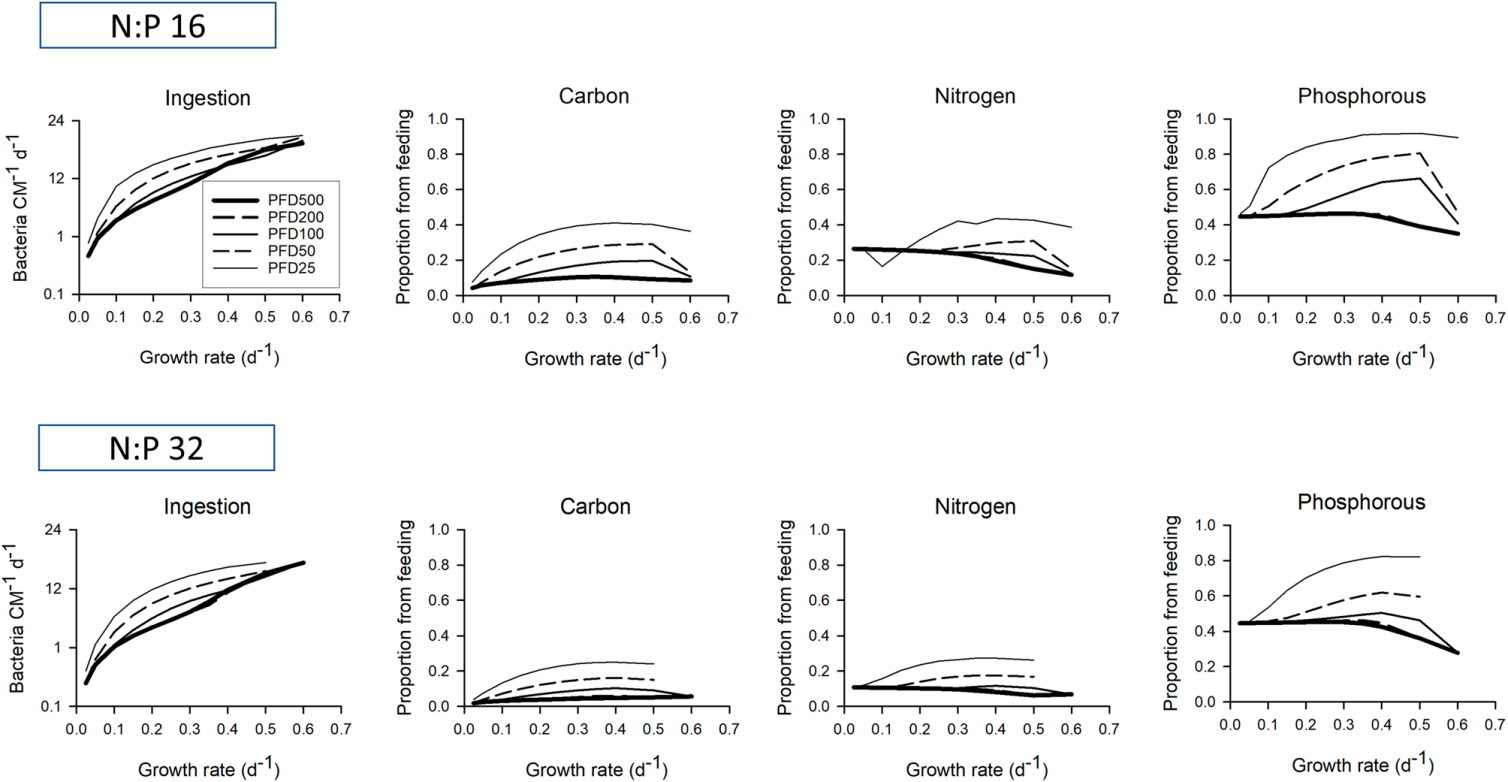
Ingestion rates and fate of ingested bacterial C, N, P biomass during mixoplankton growth at different photon flux densities. The system contained a total resource of 70 mgN m^−3^, at a mole N:P = 16 or 32, with 20% P supplied as bacteria-P. The growth rate equates to the chemostat dilution rate at steady-state. Photon flux densities (PFD) are provided as µmol photon m^−2^ s^−1^; output values for PFD200 and PFD500 are almost the same, as photosynthesis is saturated ≥ 200 µmol photon m^−2^ s^−1^. High rates of growth at some combinations of N:P and light could not be achieved. See also Figs. S7, S8.

The effect of growing mixoplankton at different photon flux densities (PFD) upon the balance of photo- versus phago- trophy is shown in Fig. 5 (cf. Fig. 4). The significance of feeding increased at lower PFD (provision of P from predation approached 100%), with significant contributions to growth at ingestions of even just a few bacteria per day for growth rates of around 0.2 d^−1^. At lower PFD, ingestion rates were higher, supporting greater contributions of bacterial C,N,P to CM biomass. These higher ingestion rates at low PFD required higher prey abundances (Fig. S7). Concurrently, with the decline in the relative importance of phototrophy at those lower PFDs, higher residual DIP was present as more P was acquired via phagotrophy. Different N:P resource levels at different PFD also affected the N and P status of the mixoplankton (Fig. S8); the N:P = 32 series had a greater level of P-stress in the mixoplankton, but for both N:P scenarios, growth at lower PFD raised the N and P status because the cells became increasingly C-limited.

**Figure 6:**
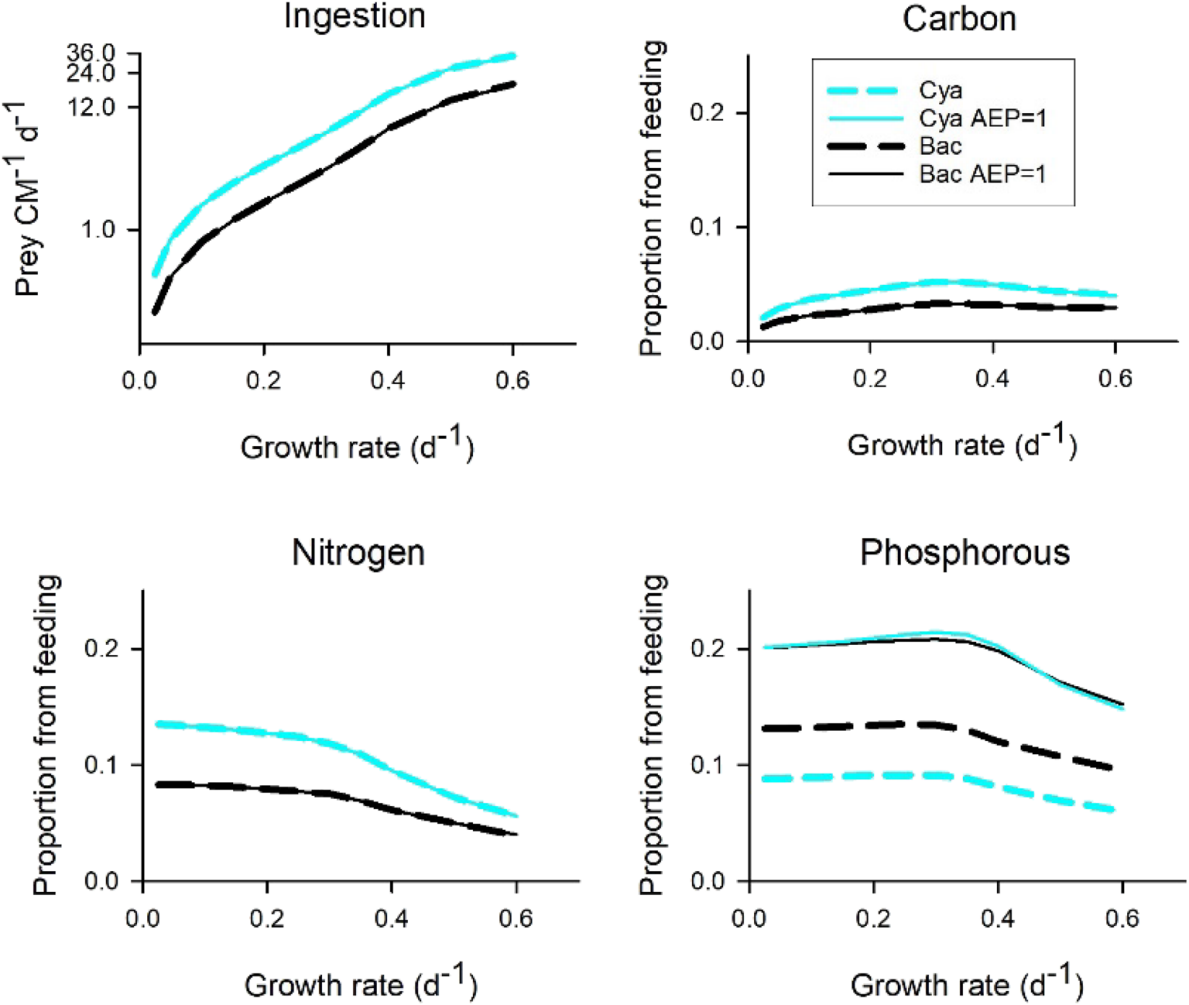
Ingestion rates and fates of ingested prokaryote of different C:N:P stoichiometry into a mixoplankton. The system contained a total resource of 70 mgN m^−3^ at a mole N:P = 16, and with 20% of the P supplied as prokaryote picophytoplankton ‘cyanobacteria’ (Cya; C:N:P of 60.6:14:1) or as bacteria (Bac; C:N:P of 29.4:7.1:1). The growth rate equates to the chemostat dilution rate at steady-state; the resource regime drives N-limiting growth of the mixoplankton. The default (dashed lines) assumes the assimilation efficiency for P is the same as N (AE_P_ = AE_N_ = 0.8); also shown are the consequences of assuming AE_P_ = 1 (AEP=1). Plots show, as 24 h averages, the ingestion rates, and the proportion of C, N and P derived from prey assimilated into mixoplankton. See Table S1 for stoichiometric values and Fig. S9 for residual prey abundance.

The simulations shown in Figs. 4 and 5 used bacterial prey. Comparisons between rates of ingestion of bacteria or cyanobacteria required to support a given rate of CM growth in the N:P = 16 resource regime are shown in Fig. 6; at most these amounted to 1 ingestion per CM per hour to make significant contributions to growth rates of up to 0.6 d^−1^. Contributions from feeding for the supply of C were low in all instances compared to contributions of N and P, noting these simulations were undertaken in a light-saturating system with 20% of resource-P supplied as prey-P (cf. Fig. 5). Ingestion rates of only 1 to 6 prey d^−1^ supported 20% of P needs at growth rates of up to ca. 0.3 d^−1^. The relative contributions from feeding declined at higher growth rates because encounter rates with prey became limiting relative to the increasing availability of inorganic nutrients (consistent with chemostat theory, and, also consistent with differences between the K_0.5_^DIN^ and K_0.5_^prey-N^ shown in Fig. 4). Prey abundances of the order of 10^4^ mL^−1^ were required to support growth rates of < 0.1 d^−1^, ranging up to ca. 5 × 10^5^ mL^−1^ of the non-motile cyanobacteria prey (‘Cya’ in Fig. S9) to support a CM growth rate of 0.6 d^−1^.

Ingestion of cyanobacteria (‘Cya’ in Fig. 6) provided a less balanced nutrition (lower P:C, Table S1) than did ingestion of heterotrophic bacteria, requiring a higher ingestion rate from a higher prey abundance (Fig. S9﻿, consistent with Fig. 3) to provide the most limiting element. A consequence was a higher contribution of ingestion to the supply of C and N from feeding on cyanobacteria. Different levels of prey-P retention (assimilation efficiency, AE_P_ = 1 versus the default AE_P_ = 0.8) only affected the proportion of P from the prey that was incorporated (see default versus ‘AEP = 1’ in Fig. 6) and did not affect prey-contributions from C or N. With a resource N:P = 32 (Fig. S10), P rather than N was the limiting nutrient with lower contributions from feeding for C and N. Consumption of smaller bacteria (‘sBac’ in Figs. S11, S12) increased the cell-specific ingestion rate required to support growth but had little other impact. Smaller mixoplankton (‘sMixo’ in Figs. S11, S12) needed to ingest fewer bacteria per cell to achieve the same contributions in terms of C,N,P (again with no impact on contributions of C,N,P as proportions of the total).

**Figure 7:**
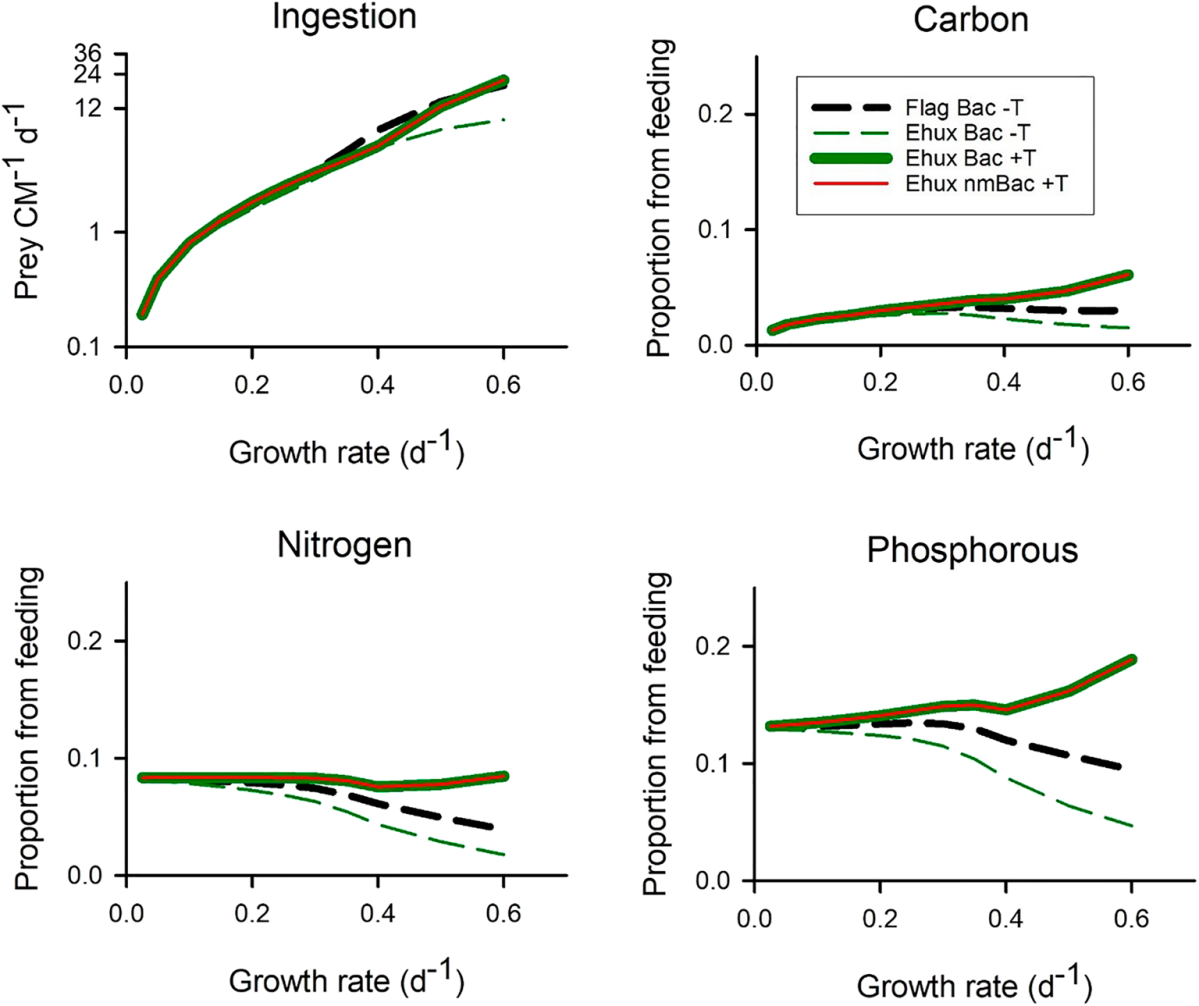
Ingestion rates and fate of ingested prokaryote biomass when the mixoplankton and/or its bacterial prey are motile or non-motile. The default is for a motile CM provided with motile bacteria prey in a non-turbulent environment (‘Flag Bac -T’); this is the same as ‘Bac’ in Fig. 6, with the same line type. The alternate CM configuration shown is for the same sized CM but as a non-motile cell, akin to *Emiliania huxleyi* (‘Ehux’). Simulations were made for ‘Ehux’ with motile bacteria in a non-turbulent environment (‘Ehux Bac -T’) or in a turbulent environment (‘Ehux Bac + T’; turbulence of 1e-3 m s^−1^), or with non-motile bacteria in a turbulent environment (‘Ehux nmBac + T’). See Table S1 for stoichiometric values and Fig. S13 for residual prey abundance.

With no turbulence, a non-motile nano-CM (hereafter, ‘Ehux’) was less phagotrophic than its motile nano-CM counter-part at growth rates above ca. 0.2 d^−1^ (Fig. 7), requiring a higher bacterial abundance to support a given ingestion rate (Fig. S13). Even so, the phagotrophic contribution towards Ehux growth was still significant at ingestion rates of just a few bacteria per day (Fig. 7). Inclusion of turbulence greatly increased encounter rates and was more important than prey motility for supporting the encounters that would lead to phagotrophy (Fig. 7, consistent with Fig. 2b). Turbulence thus increased the proportion of prey biomass contributing to mixoplankton growth and decreased the prey abundance level required to support ingestion to typically < 10^4^ mL^−1^ even for growth rates of 0.4 d^−1^ in the test regime (Fig. S13, consistent with Fig. 2b).

## Discussion

### Synergism between phototrophy and phagotrophy

Simple allometric-stoichiometric analyses (Figs. 2, 3, S4-S5) suggest that grazing rates of only a few prey per day would not supply significant amounts of N or P nutrition. Even for the smaller nano-CM species (< 5 µm ESD; e.g., *Florenciella* sp., *Mantoniella antarctica*, *M. squamata*^11^) the contribution at such low grazing rates appears low, noting that high grazing rates upon bacteria have been measured in these organisms (Table S3). However, this analysis takes no account of the co-operativity between phototrophy and phagotrophy (Fig. 1) that underpins mixoplankton physiology. The analysis also assumes a need to support the maximum mixoplankton (Redfield-like) C:N:P. In reality, phototrophic growth continues at much lower N:C and (especially) P:C^28^, and hence would be supported by nutrients obtained at lower predation rates than indicated. Simple allometric-stoichiometric analyses thus provide only a broad overview of allometric CM-prey relationships against which to interpret the results from simulations made using the mechanistic simulation model.

Although the simple stoichiometric-allometric analysis indicated that grazing at low rates is insignificant (providing < 5% of N or P), a deeper consideration of physiology linked to variable stoichiometry and interactions between phototrophy and phagotrophy indicates otherwise (Fig. 4 onwards). Interactions between phototrophy and phagotrophy resulted in an emergent enhancement in resource affinities. Low half-saturation constants for resources flag an enhanced competitive advantage at low resource concentrations. Residual resource abundance at µ = µ_max_/2 (i.e., K_0.5_) were of the same order for DIP and prey-P (i.e., K_0.5_^DIP^ ≈ K_0.5_^preyP^) when both were equally available in the resource pool (Fig. 4a, c; for reference, 1 mgP m^−3^ ≡ 0.03 µM DIP). For N, however, K_0.5_^DIN^ < K_0.5_^preyN^ (Fig. 4b, c). These results indicate that phagotrophy for N would be of greatest value at high prey abundance, such as in coastal waters in post-spring-bloom conditions (e.g., predation rates of 192–432 bacterial cells CM^−1^ d^−1^ by *Haptolina ericina*^19^) while P acquisition by phagotrophy would be of especial value under oligotrophic conditions^29^. Importantly, mixoplanktonic activity decreases the value of K_0.5_ for both the inorganic nutrient and for prey (in terms of both N and P) compared to resource acquisition using either resource alone (Fig. 4a, b, c), as would be the case for phytoplankton or protistan-zooplankton, respectively. This supports arguments for a competitive advantage for mixoplankton in different niches^25,30^, and of the importance of considering mixoplanktonic activity as synergistic rather than simply phototrophy plus phagotrophy^31^.

At lower light, C ingestion becomes increasingly important (Fig. 5) with consequences also for the acquisition of the other elements (Figs. 5, S7, S8) that are brought in together with C in the food package and then subjected to catabolic and anabolic processing (Fig. 1). Selection pressure for a high photosynthetic capability in mixoplankton growing in coastal waters, with higher bacteria prey abundance, may be expected to be lower than for off-shore species. Phagotrophy at high prey abundance may even suppress phototrophy, with the coastal CM *Ochromonas* sp. exploiting phagotrophy over phototrophy, and the opposite for off-shore *Ochromonas* sp. in a low prey ecosystem^32^. As phagotrophy is the ancient trait^33^, retention of chloroplasts for mixotrophic autotrophy may have been selected for as a competitive advantage to compensate for inadequacies in phagotrophy to supply C in oligotrophic conditions, rather than phagotrophy to compensate for light-limitation of phototrophy in coastal waters.

We assumed that the ability of the prokaryote prey to acquire resources from even very low levels^5^ would enable them to maintain a good C:N:P status^34^, though that is not necessarily always so^35,36^. The relative contribution of prey-P to CM growth was always greater than that of prey-N (Figs. 4, 5, 6, 7). Retention efficiencies of each element vary as functions of digestion (with assimilation efficiency, AE) and metabolism synthesising new biomass. For a heterotroph, this specific dynamic action (SDA) inevitably leads to the loss of a portion (ca. 30%)^37^ of the C with an allied stoichiometric loss of other elements and especially of N. For mixoplankton, however, SDA may be effectively zeroed because the input of new C from photosynthesis can compensate for respiratory losses and also enables immediate reassimilation of N and P lost by SDA from phagotrophy. Concurrently, this activity decreases the demand for externally supplied inorganic nutrients. As mixoplankton have immediate access to ammonium from digestion of prey-N (Fig. 1), they will have decreased demands for nitrate assimilation compared to co-occurring phytoplankton, and thence further save energy and demand for Fe^38^.

### Significance of low grazing rates

It is unlikely that grazing on prokaryotes in nature would alone provide the sole source of N or P, not least because there are invariably detectable levels of DIN and DIP; for this reason most of our simulations focussed on providing the bulk of N and P resources in inorganic forms (Figs. 5, 6, 7). Even here, and in contrast to the implications of the simple stoichiometric-allometric analysis (Figs. 2, 3), ingestion of just a few prokaryotes per day made a significant contribution to nutrient supply to the CM, consistent with the results of^39^. Most ingestion rates from the simulations were ca. 2–4 prey cells d^−1^ at growth rates of around 0.35 d^−1^ providing ca.15% of the limiting nutrient from a resource containing 20% P as prey. Such rates can provide more than just a survival mechanism^19^. Ingestion rates of 1–2 prey h^−1^ could supply 100% of P for this growth rate (Fig. 4c, e). A minimum critical support of growth at ca. 0.03 d^−1^ (to overcome mixing losses^27^) was achievable at ingestion rates of ca. < 1 prey d^−1^ (Figs. 6, S10). To put these results into perspective, consumption of ca. 0.5–5 prokaryote CM^−1^ d^−1^ has been observed in coccolithophorids^17^ *Calyptrosphaera sphaeroidea*, *Calcidiscus leptoporus*, the haptophyte *Phaeocystis globosa*^18^ and the cryptophyte *Teleaulax amphioxeia*^20^. Table S3 collates ingestion rate data for various mixoplankton-prokaryote combinations; the maximum ingestion rates observed in similar sized mixoplankton-prey combinations to those simulated (ca. 100–150 prey d^−1^) are consistent with ingestion rates at half maximum growth rate on a 100% resource prey diet (Fig. 4e). While an underlaying understanding of the organism’s ecophysiology that explains the range of those data is missing^7^, we must expect mixoplankton to adapt to different environments (high vs low availability of light, nutrients and/or of prey) by optimising their potential for phagotrophy and phototrophy^40^.

The globally important CM, *Emiliania huxleyi*, typically grows in turbulent waters, appearing well adapted to life in low nutrient systems^41^. The recent observation that it can eat bacteria^17^ may help explain their competitive edge. Our simulations show that consumption of just 1 prokaryote prey d^−1^ into a cell type like *E. huxleyi* can at the least support a basal growth rate (i.e., 0.03 d^−1^; Fig. 7), and ingestion of several prey per day can make significant contributions to the supply of P. Prey abundance < 10^5^ mL^−1^ could support ingestion rates of 1–12 per day, especially with turbulence (Fig. S13), and indeed turbulence appears to have an important potential role in enabling mixoplankton-prey encounters (Fig. 7). This situation would change if stratification became more common under climate change, favouring more motile mixoplankton.

Various uncertainties exist over the calculation of these grazing rate values, especially as they assume a homogenous distribution of edible prey. However, motile bacteria are often attracted to the phycosphere of phototrophic protists by the leakage of organics^42^, while many prokaryotes form aggregations especially under turbulence^43^, or in the presence of grazers^44^. Cell per cell encounter rates may thus be higher than assumed from average bulk homogenous cell abundances. Efficiency of capture and thence ingestion upon encounter is certainly less than 100%; here we have assumed only a 20% success rate^45^. Nonetheless, set against the abundance of oceanic prokaryotes^4,6^, prey levels to support ingestion rates of 1–24 prey cells d^−1^ are quite plausible.

To close the nutrient cycle, there is every reason to suspect that in nature, any nutrients (including DOC from phototrophy, and debris from digestion) voided by the mixoplankton will be accessed by bacteria, and that some of those bacteria will be subjected to predation by mixoplankton. This relationship has been viewed as akin to mixoplankton farming the prokaryotes^15^, rather than competing for common resources^46^. The timing and significance of such events during plankton succession provides an additional level of interplay between primary production and bacterial production^47^.

### Implications for field work and ecology

The importance of the contributions of C, N, P from feeding were inversely proportional to the stoichiometric needs of the mixoplankton (Figs. 4, 6, 7). Consuming prey is of greater benefit in poor inorganic nutrient regimes^48,49^, providing a resource combination coupled with phototrophy in mixoplankton this is unavailable to either of their phytoplankton and zooplankton competitors. This advantage for mixoplankton is greater again in consequence of the interaction between nutritional routes that decreases K_0.5_ for both inorganic and prey resources (Fig. 4). As a result, mixoplankton could capitalise on the emergence of opportunities in consequence of the prior grazer or viral control of competitors^50^, helping to define their ecological niche^25,30^.

Simulations show that contributions to the total C from feeding is ca. 10% (Fig. S6); this is similar to the loss rates of C as DOC from phototrophic plankton^51,52^. Measuring feeding using ^14^C ﻿methods would thus likely fail to flag the importance of phagotrophy, the signal being lost in data noise. Other isotopes to track ingestion rates have been used^29^, but more commonly organism counts are used to follow plankton feeding dynamics^53^. However, low grazing rates by mixoplankton on prokaryotes, shown by the simulations as significant, may go undetected given difficulties in measuring such events in nature^54,55^. Accordingly, we suggest that if there is documented evidence that a given phototrophic protist plankton species is capable of performing phagotrophy^11,16^, then that organism should be considered as a functioning constitutive mixoplankton in nature. Ignoring this potential would lead to an under-appreciation of mixoplanktonic activity in ecology^56^.

## Conclusion

Synergism between phototrophy and phagotrophy in constitutive mixoplankton results in low prey ingestion rates having a significance beyond that which is immediately apparent, especially in studies focussed only on carbon. This contribution of low ingestion rates should be viewed positively for primary production by mixoplankton and not as a substitute for photosynthesis; the supply of non-C nutrients stimulate C-fixation in oligotrophic settings. Climate change promotion of the success of prokaryote plankton^5,6^ can act as a vector for nutrients for mixoplankton and thence onwards to higher trophic levels. We may thus expect to see an increased ecological role for constitutive mixoplankton, exploiting the increased abundance of their prey set against a decreased concentration of inorganic nutrients.

## Methods

For brevity, we use the term ‘bacterivory’ as a general term for consumption of pico-prokaryotic prey and not just with reference to bacteria.

### Stoichiometric & allometric analysis

To explore whether low rates of grazing could be significant for constitutive mixoplankton (CM), we considered the stoichiometric and allometric relationships between the CM predator and their prey (affecting the value of the food package), as affected by their motilities and by turbulence (promoting predator–prey encounters). Transforms from protist, bacteria and cyanobacteria cell size to their respective C content, and C:N:P stoichiometries (Supplementary Methods) were used to provide a first estimate of the most optimistic value of low ingestion rates, assuming a 100% efficiency for capture and assimilation to support a growth rate of a doubling per day. This analysis was also used to couch the selection of conditions used for simulations.

### Simulation model configuration

The potential role of phagotrophy coupled with phototrophy on CM growth was explored in detail using a system dynamics simulation modelling approach^15,57^. The model provides a dynamic, acclimative, variable C:N:P stoichiometric description capable of resolving growth exploiting phototrophy and phagotrophy consistent with the synergistic physiological processes^28,31^ (Fig. 1; see also Supplementary Methods). Internal recycling of inorganic nutrients from prey digestion was exploited in preference to external inorganic sources supporting phototrophy; this activity negates losses normally associated with specific dynamic action (SDA) during prey digestion^37^. A proportion of ingested material was voided by mixoplankton (defined as 1-AE, where AE is the assimilation efficiency); the value of AE with good quality prey (i.e., prey C:N:P similar to that of the consumer) was set as 0.8^58^. We considered two contrasting scenarios for AE of phosphorous: (i) where assimilation of P followed that of N (i.e., AE_N_ = AE_P_ = 0.8), and, (ii) the extreme, where all ingested P was retained (i.e., AE_P_ = 1).

From empirical evidence of the importance of bacterivory for nano-sized CM species^20,24,25,32,39^, and based on the results from our initial stoichiometric-allometric studies, the model was configured for a motile CM cell, with equivalent spherical diameter (ESD) of 5 µm and a maximum growth rate of a doubling per day (µ_max_ = 0.693 d^−1^). Predator–prey encounters are affected by motility of both organisms, and by turbulence^59,60^; by default, no turbulence was provided, and the mixoplankton were motile. Bacteria were configured as motile or non-motile, while prokaryote picophytoplankton were non-motile. Motility, as appropriate, was allometrically related to cell size, with 20% of CM encounters with prey resulting in capture and ingestion^45^. We assumed that the prokaryote prey, of 1 µm ESD, would be sufficiently adept at resource acquisition^5^ such that they maintained optimal stoichiometry^34^. Accordingly, we set C:N:P by mass for bacteria at 29.4:7.1:1, and for prokaryote picophytoplankton at 60.6:14:1 (Table S1).

### Simulation scenarios

Simulations for most scenarios were run with a photon flux density (PFD) of 200 µmol m^−2^ s^−1^, in a light:dark ratio of 16 h:8 h; this irradiance saturates phototrophy in the model and can support growth at the maximum rate. To study the implications of nutrition for the mixoplankton in isolation of other processes linked to prey physiology, we ran the simulations in a chemostat-like scenario (Fig. ﻿S3), with nutrition supplied as dissolved inorganics (ammonium and phosphate; DIN and DIP) and as non-growing prey. These resources were supplied with a total N concentration of 70 mgN m^−3^ (≡ 5 µM if supplied 100% as DIN). Resource P was allocated between DIP and prey prokaryote-P; the allocation of N between DIN and prokaryote-N was then established by reference to the N:P stoichiometry of the prokaryote prey (as indicated above). Impacts of photo-phago-trophy under different nutrient and prey concentrations were explored using simulations under potentially N-limiting (resource molar N:P = 16) and P-limiting (N:P = 32) conditions. The default partitioning of P between DIP and prey-P was 80:20; this provided a low prey abundance consistent with our interests in testing the significance of only few prey ingestion events. It is worth noting that routine oceanographic measurements of P are restricted to DIP.

In a chemostat at steady-state with the dilution rate at µ_max_/2, the residual (i.e., remaining) concentration of the limiting resource is the half saturation constant for growth^61^, K. Growth of the mixoplankton was forced to half the maximum rate (i.e., steady-state growth with dilution = 0.35 d^−1^) and the proportion of P supplied at bacterial-P varied between 0 and 100% (Table S2), with saturating light (200 µmol photon m^−2^ s^−1^). Other simulations used a range of irradiance values, from growth limiting PFD of 25 µmol photon m^−2^ s^−1^ to growth saturating PFD of ≥ 200 µmol photon m^−2^ s^−1^. With different resources and a complex multi-nutrient interaction between phototrophy and phagotrophy, played out during growth in a light–dark cycle, we refer to the values of residual resources in chemostat systems run at a dilution rate of µ_max_/2 (i.e., here, at a rate of 0.35 d^−1^) as K_0.5_ values (e.g., K_0.5_^DIP^ and K_0.5_^preyP^ for DIP and prey P, respectively).

To consider predation on smaller prey and by a smaller predator, as projected with climate change^6,62^, we tested CM feeding on smaller bacteria and also feeding by smaller CM cells. For the former, bacteria were configured at 0.8 µm ESD (rather than 1 µm), and for the latter CM were configured at 4 µm ESD (rather than 5 µm). We also explored the implications of turbulence on feeding by a non-motile CM (e.g., *Emiliania huxleyi*; see ^17^ and references therein).

For reference, natural prey abundances for prokaryotes are typically (depending on season and location) around 10^4^–10^6^ mL^−1^ ^6,63^.

## Supplementary Information

Supplementary Information.

## Data Availability

The model description, configuration and data that support the findings of this study are available in the supporting information associated with this article.
