# Supplementary material for: Low rates of bacterivory enhances phototrophy and competitive advantage for mixoplankton growing in oligotrophic waters
Source: Sci Rep. 2023 Apr 27;13:6900. doi: 10.1038/s41598-023-33962-x (PMC10140275; doi:10.1038/s41598-023-33962-x)
Supplement: Supplementary file 1 — Supplementary Information. [file 41598_2023_33962_MOESM1_ESM.pdf]

SUPPLEMENTARY INFORMATION

Low rates of bacterivory enhances phototrophy and competitive advantage for mixoplankton growing in oligotrophic waters

Aditee Mitra, Kevin J Flynn

Supplementary Methods

Stoichiometric and allometric analysis

To explore whether low rates of grazing could be significant for nano-CM, we conducted a stoichiometric and allometric analysis. The contribution of phagotrophy to growth is a function of the following:

i. *Stoichiometric and allometric relationships between the CM predator and their prey*: The former sets the value (quality) of the food package, whether it is relatively high or low with respect to the elemental composition. Allometry affects the food density of the package, whether different prey, of a given size, have more or less nutrients (e.g., C,N,P) per cell.

ii. *Motilities of the CM-predator and their prey*: These, together with the sizes of the organisms, affect predator-prey encounter rates. Turbulence also greatly increases those encounter rates.

CM growth rate: This sets the demand rate for resource acquisition, including the feeding rate. The default expectation for phototrophic plankton is for their growth rate to be around a doubling per day (i.e., 0.693 d^{-1}, Nelson and Brand, 1979). This expectation is associated with nutrient replete immature systems. In oligotrophic mature systems, mixoplankton likely grow at slower rates; their feeding demands would thus be pro rata less than for a cell with a growth rate of 0.693 d^{-1}.

For the mixoplankton predator, we related C-biomass to cell size using the equations of Menden-Deur and Lessard (2000), and rates of motility to Flynn and Mitra (2016). For the bacteria and the picophytoplankton prey, we related C-biomass to cell size using information in Romanova and Sahzin (2010) and Heywood et al. (2006), respectively. Heterotrophic bacteria were assumed to be motile (Johansen et al., 2002), and picophytoplankton non-motile. Encounter rates were computed using the equation of Rothschild and Osborn (1988), with

reference to sizes of the organisms (expressed as equivalent spherical diameter, ESD), and their motilities together with turbulence which together increase encounter rates. We assumed mixoplankton C:N:P stoichiometries (mass ratio of ca. 6.1:4.8:1) according to the phytoplankton analysis conducted by Geider and LaRoche (2002), noting that many flagellate ‘phytoplankton’ are actually mixoplankton (Unrein et al., 2014; Zubkov and Tarran, 2008; Leles et al., 2019). Bacteria C:N:P stoichiometries were obtained from Heldal et al. (1996), Tortell et al. (1996) and Zimmermann et al. (2014), and those for picophytoplankton from Bertilsson et al. (2003) and Cunningham and John (2017). Together, these references evidence differences in the C-density of bacteria and the prokaryote phytoplankton (Table S1), such that the latter are ca. 10-15% denser in terms of C and N, but contain half the amount of P.

We calculated the number of prokaryote cells required to be assimilated per CM predator cell per day to provide, at the extreme, all the structural C, N and P needed to support a doubling per day (i.e., for a growth rate of 0.693 d^{-1}). To place this in context we have identified those combinations of prey and predator that could achieve that nutritional support through ingesting (on average) just 1 prey item per day, or 1 prey per hour. If we then consider combinations of predator and prey allometry and stoichiometry that support a growth rate of 0.693 d^{-1} at an ingestion rate of 1 prey h^{-1}, those same combinations, but at an ingestion rate of 1 prey d^{-1}, would support a growth rate of (0.693/24 =) 0.029 d^{-1}; this value aligns with the minimum growth rate needed to compensate for a loss rate of the CM through mixing from the sunlit mixed layer into the meso-pelagic zone (e.g., Fasham et al., 1990). These calculations provide the most optimistic case, with a 100% efficiency for capture and assimilation to attain the maximum growth rate. In reality, the interactions between the different trophic modes are complex and intertwined (see Fig. 1), and growth rarely occurs at the maximum rate. To obtain an enhanced, more realistic appreciation of the potential for low rates of grazing requires the use of a mechanistic simulation model.

Simulation Model

The potential role for CM growth provided by phagotrophy upon prokaryotes coupled with phototrophy was explored using a simulation model developed from our earlier models (Flynn and Mitra, 2009; Mitra et al., 2021; Flynn and Mitra, 2023). These models have been deployed in various scenarios (Leles et al. 2018; Lin et al., 2018; Leles et al., 2021; Li et al., 2022). Our models are the only mixoplankton models to date that have been subjected to ‘expert witness validation’ (hence the authorship list in Mitra et al., 2014, 2016), and tuning to empirical data

(Lin et al., 2018; Li et al., 2022). The model thus conforms to aspirations for a digital twin description of these organisms (see Flynn et al., 2022).

The task at hand demands a comprehensive plausible description of C,N,P physiology, operating in an explicit light-dark cycle. Our mixoplankton models are the only ones to deliver to that need; the physiological system is complex with various layers of feedback such that gross simplifications will only introduce uncertainties.

The model is capable of resolving growth exploiting phototrophy, osmotrophy and phagotrophy (Fig. 1; Mitra et al., 2021). It provides a full variable stoichiometric (C,N,P,Chl) description in which the simulated growth dynamics are modulated by feedback processes in line with the physiological acclimation processes that occur in reality. The model simulates the use of different nutrient types (inorganic and organic) under different illumination regimes. Internal nutrient recycling, impacted by different supply and demand, and anabolic / catabolic respiration rates varying over the light:dark cycle, are described. There is an explicit inclusion of allometry for consideration of predation, and of the biomass allocation to photosystems (Fig. S1). The model features extensive modulation between submodules describing facets of the physiology, with functioning in keeping with biochemical understanding as described using a coarse-grain systems biology approach.

The model was constructed and run within Powersim Studio 10 software (Powersim Software AS, Norway), under Euler integration with a step size of 0.015625d. An example of the model operating in batch-culture mode, exploiting two different prey organisms, nitrate, ammonium and phosphate, is given in Fig. S2.

Functional Equations

The following provides a functional-equation description of the model as text strings, with the form:

result = *f*{comma delimited list of factors involved in deriving the result}

Underlined terms in the equations are rates. Those in bold donate terms that provide a positive interaction (i.e., the result increases when the term increases; these are usually enacted via a curvi-linear function); terms not in bold may involve negative or more complex interactions (such as bell-shaped for prey allometry affecting capture).

The equations are provided working backwards from the emergent organism growth rate, with descriptions of the steps enabling that rate to be attained.
NOTE: For brevity, reference to state variables does not include 'prot'.

Total protist biomass C is given as, $^{T}C = {}^{M}C + {}^{PM}C + {}^{C}C$.

State variables

The model comprises the following state variables and associated flows:

Core structure: prot^{C}C; mgC m^{-3}
d prot^{C}C/dt = {anabolism} – {catabolism when ^{M}C:^{T}C is critically low}

Metabolic pool of C: prot^{M}C; mgC m^{-3}
d prot^{M}C/dt = {osmotrophy(C)} + {phagotrophy(C)} + {phototrophy} + {degradation of prot^{PM}C} – {synthesis of prot^{PM}C} – {anabolism} – {catabolism} – {NO3-assimilation(reductant)} – {DOM(C)-leak}

C in chloroplasts: prot^{PM}C; mgC m^{-3} (this sets the maximum rate of photosynthesis, Pmax)
d prot^{PM}C/dt = {synthesis of ^{PM}C} – {degradation of ^{PM}C}

Photopigments: protChl; mgChl m^{-3}
d protChl/dt = {synthesis of Chl} – {degradation of Chl}

Organism-P: protP; mgP m^{-3}
d protP/dt = {osmotrophy(P)} + {phagotrophy(P)} + {DIP-assimilation} – {P-regeneration} – {DOM(P)-leak}

Organism-N: protN; mgN m^{-3}
d protN/dt = {osmotrophy(N)} + {phagotrophy(N)} = {NH4-assimilation} + {NO3-assimilation} – {N-regeneration} – {DOM(N)-leak}

Depending on the application, additional state variables can be included:

- **protCells** (cells m^{-3}): cells, required for a dynamic description of cell-size with nutrient status, diel light cycle and temperature
- **protSi** (mgSi m^{-3}); organism-Si, required for diatoms
- **protANA** (mgNA m^{-3}); acquired nucleic acid material from phototrophic prey, required to support acquired phototrophy in plastidic specialist non-constitutive mixoplankton (pSNCM)

Growth and nutrient status

Ultimately growth is a function of the nutritional status of the organism (in terms of elements C, N and P) and the maximum growth rate potential. The latter varies with temperature, T, around the value of μ_{max} at a reference temperature, μ_{maxRT}.

growth = *f*{**C-status**, **N-status**, **P-status**, **μ_{max}**, losses}

μ_{max} = *f*{**μ_{maxRT}**, **T**}

The nutrient status defines the health of the organism in terms of C (^{M}C:^{T}C), N (N:^{T}C) and P (P:^{T}C), and is a function of various inputs and outputs. Inputs are associated with the use of dissolved organic substrates via osmotrophy, prey via phagotrophy, and also the use of inorganics via phototrophy. Losses occur through respiration and regeneration, and also through the leakage of metabolites as dissolved organic matter (DOM), some of which may be recovered via osmotrophy (Fig. S1).

C-status = *f*{**osmotrophy**, **phagotrophy**, **phototrophy**, C-respiration, DOM-leak}

N-status = *f*{**osmotrophy**, **phagotrophy**, **phototrophy**, **DIN assimilation**, N-regeneration, DOM-leak}

P-status = *f*{**osmotrophy**, **phagotrophy**, **phototrophy**, **DIP assimilation**, P-regeneration, DOM-leak}

Losses = *f*{**C-respiration**, **N-regeneration**, **P-regeneration**, **DOM-leak**}

Growth is associated with catabolic (including basal) and anabolic respiration, part of which is associated with specific dynamic action (SDA) during prey digestion and assimilation. Anabolic respiration is affected by the flows of resources via the different trophic mechanisms. Nitrate assimilation incurs an additional cost for reduction of nitrate to nitrite to ammonium.

There are also losses of C, N, P required to preserve organism stoichiometry within the bounds of acceptable C:N:P.

C-respiration = f{**μ_{max}**, **basal respiration**, prot-C:N, **C-assimilation**, **NO3-assimilation**}

C-assimilation = f{**osmotrophy**, **phagotrophy**, **phototrophy**}

N-regeneration = f{**C-respiration**, **prot-C:N**, **prey-C:N**, **digestion**, SDA}

P-regeneration = f{**C-respiration**, **prot-C:P**, **prey-C:P**, **digestion**, SDA}

DOM-leak is closely associated with osmotrophy (see further below).

Cell division occurs when the cell reaches a critical size (which varies with nutrient status and temperature affecting the growth rate), and typically occurs in phototrophs within a specific part of the diel light:dark (LD) cycle.

division = *f*{**size**, critical size, LD}

critical size = *f*{T, C-status, N-status, **P-status**, **growth**}

The size of the organism affects predation for phagotrophy, and whether it itself is likely to encounter its own predator.

Osmotrophy

Osmotrophy depends on the concentration of the substrate, [DOM], the C:N:P status of that material, and the uptake kinetics parameters of the maximum uptake rate (${}^{DOM}V_{max}$) and the substrate affinity (i.e., the reciprocal of the half saturation constant, K_{DOM}). The uptake kinetics depend on the nutrient status of the organism; cells that are nutrient-stressed have a higher uptake potential and a high affinity.

osmotrophy = *f*{**[DOM]**, DOM-C:N:P, **${}^{DOM}V_{max}$**, **$1/K_{DOM}$**}

${}^{DOM}V_{max}$ = *f*{C-status, N-status, **μ_{max}**}

$1/K_{DOM}$ = *f*{C-status, N-status}

Against the gains from osmotrophy there are losses with the leakage of DOM. At especially high growth rates, which require a high nutrient status and hence a replete the internal metabolite pool containing mM concentrations, DOM inevitably leaks. Osmotrophy may

recover some of that leakage. The net leakage of N-containing DOM (as amino acids) is most significant during N-replete growth conditions, while leakage of DOC (sugars) occurs especially with high rates of phototrophy, including when N becomes exhausted and the cell has yet to down-regulate photosynthesis.

DOM-leak = f{C-status, N-status, **osmotrophy**, **phagotrophy**, **phototrophy**, **μ_{max}**}

Phagotrophy and voiding

Phagotrophy brings in resources from the assimilation of prey biomass; note the plural in prey-assimilations. Prey need to be encountered (which depends on the sizes of the predator organism and of the prey, their respective motilities and turbulence), captured (which like the predator motility varies with satiation, and also with the 'taste' of the prey as affected by its stoichiometric quality) and ingested. These processes are prey-species specific; the collective biomass from many ingestions, perhaps of different prey organisms, is then digested. During digestion a fraction of the ingested prey is subjected to voiding (depending on the assimilation efficiency, AE, predator satiation and the food quality), and another fraction is lost associated with specific dynamic action (SDA) as the prey biomass is subjected to catabolic and then anabolic processes. The internal recycling of regenerated inorganic nutrients is a critical step in mixoplankton (Fig. S1; see *Inorganic nutrient assimilations*, below).

phagotrophy = *f*{**prey-assimilations**}

prey-assimilation = *f*{**digestion**, SDA}

digestion = *f*{**ingestion**, voiding, **prey C:N:P**, **μ_{max}**}

ingestion = *f*{**capture**, **μ_{max}**}

capture = *f*{C-status, N-status, P-status, **prey quality**, prey allometry}

encounter = *f*{**[prey]**, allometry, **motility**, **prey motility**, **turbulence**}

motility = *f*{C-status, N-status, P-status}

voiding = *f*{**ingestion**, **C-status**, **N-status**, **P-status**, prey quality, AE}

Phototrophy

Photosynthesis depends on light, the availability of dissolved organic C (DIC, especially as CO_2 and HCO_3^-), photopigment content (Chl:C), the value of alpha governing the initial slope of the light-photosynthesis curve, and the maximum rate of C-fixation (P_{max}). The value of P_{max} is set by the size of ^{PM}C:^{T}C. For organisms with a constitutive ability to photosynthesise, both Chl:C and P_{max} are modulated by the demand for C and energy, reflected by the organisms' nutritional status and growth rate potential. For the non-constitutive mixotrophs (NCM), phototrophy is acquired from captured phototrophic prey. Light is a function of the photon flux density at the water surface and of attenuation within the water (which varies with the biomass of the pigmented organisms). Light also varies over the diel light:dark cycle; this imparts a diel cycle on phototrophy that then feeds through to affect osmotrophy and phagotrophy.

phototrophy = *f*{light, **[DIC]**, **Chl:C**, **alpha**, **P_{max}**}

Chl:C = *f*{C-status, **N-status**, (for NCM, prey-Chl:C, capture)}

P_{max} = *f*{C-status, **N-status**, P-status, **μ_{max}** (for NCM, prey-P_{max}, capture)}

Inorganic nutrient assimilations

Inorganic nutrients are sourced both internally, as regenerative products of prey assimilation, and externally; use of the former takes priority and will be affected by prey C:N:P. The use of external nutrients depends on the substrate concentrations ([DIP], [NH4], [NO3]) and the respective uptake kinetics (uptake-Vmax, affinity). The latter vary with the nutritional state of the organism, with uptake potential enhanced when nutrient-stressed and, at the extreme, shut down if nutrient-replete (i.e. uptake-Vmax tends to zero at elevated nutrient status).

DIP assimilation = f{prey-assimilation, prey C:N:P, SDA, **[DIP]**, **$^{DIP}V_{max}$**, **$1/K_{DIP}$**}

$^{DIP}V_{max}$ = f{P-status, **μ_{max}**}

$1/K_{DIP}$ = f{P-status}

The uptake of DIN is affected also by the P-status of the organism. The uptake kinetics for ammonium (NH4) provide for development of a much enhanced uptake capability over that for nitrate (NO3), with that development also commencing at a higher N-status. The latter results in ammonium being taken up 'in preference' to nitrate (there is no 'inhibition' term

controlling NO3-assimilation by [NH4]); if the supply of ammonium from internal recycling plus external sources cannot meet the demand, then the ability to use nitrate is de-repressed.

DIN assimilation = f{prey-assimilation, prey C:N:P, SDA, **NH4-assimilation**, **NO3-assimilation**}

NH4-assimilation = f{**[NH4]**, $\mathbf{^{NH4}V_{max}}$, $\mathbf{1/K_{NH4}}$}

NO3-assimilation = f{**[NO3]**, $\mathbf{^{NO3}V_{max}}$, $\mathbf{1/K_{NO3}}$ }

$^{NH4}V_{max}$ = f{N-status, P-status, $\boldsymbol{\mu_{max}}$}

$1/K_{NH4}$ = f{N-status}

$^{NO3}V_{max}$ = f{N-status, P-status, $\boldsymbol{\mu_{max}}$}

$1/K_{NO3}$ = f{N-status}

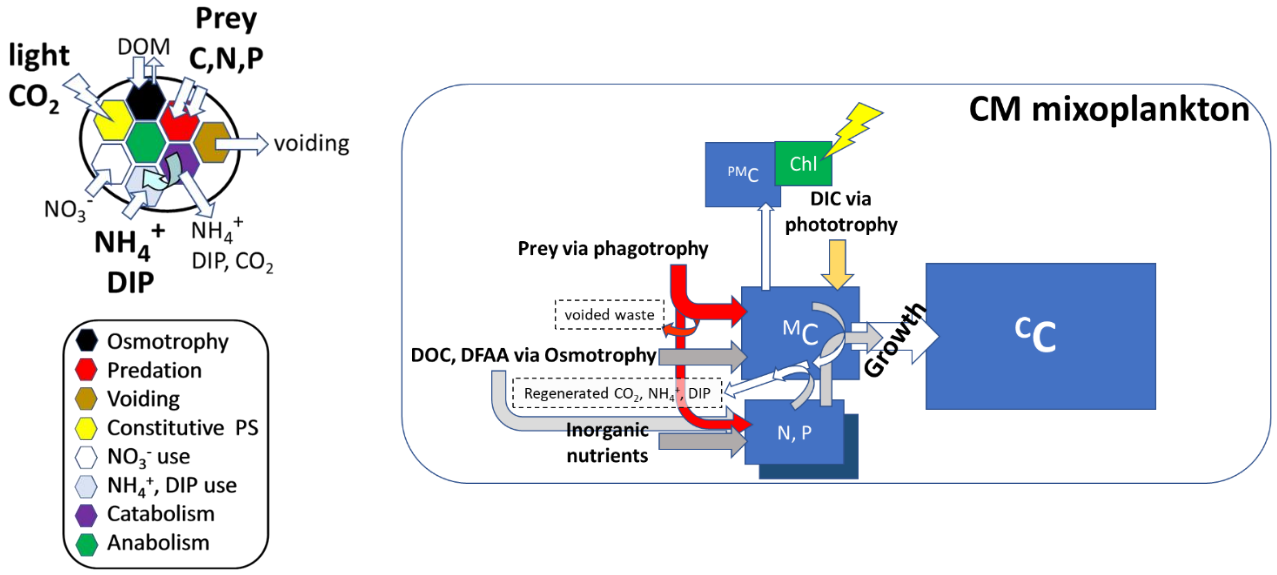

Fig. S1. Schematic of the modular structure of protoDRAMA (left) and (right) interactivity between the state variables for structural C (^{C}C), metabolite C (^{M}C), N and P biomass, C-biomass allocated to the photosystem (chloroplast; ^{PM}C), and chlorophyll (Chl).

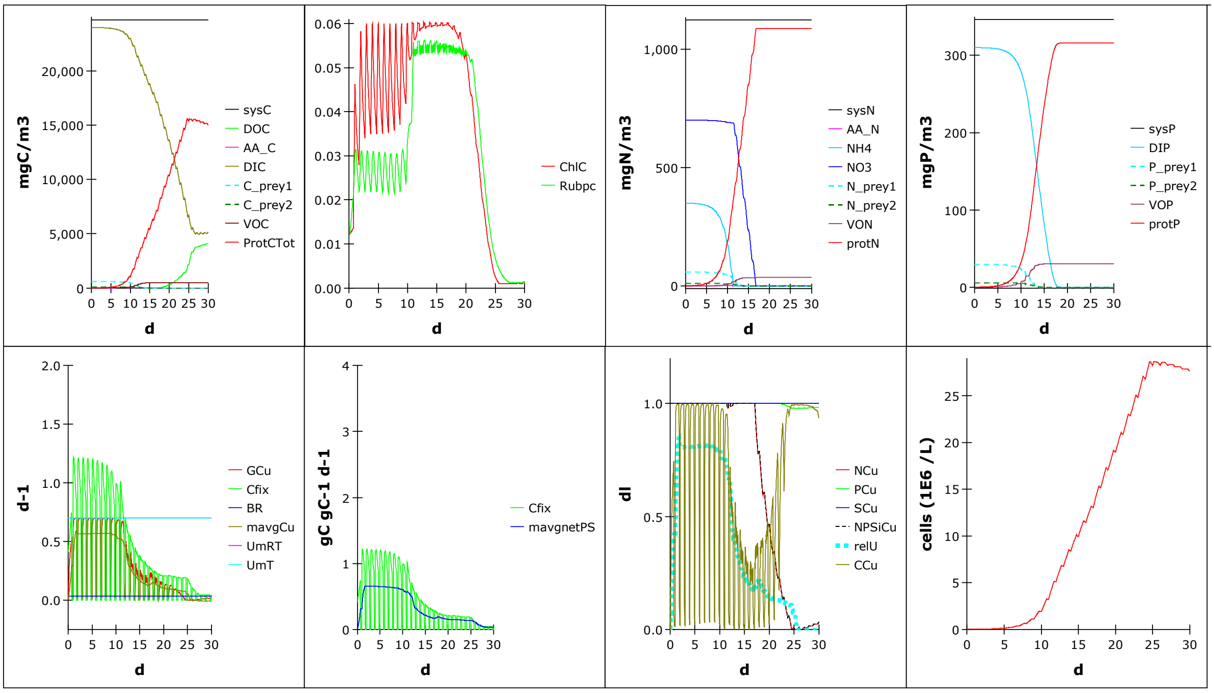

Fig. S2. Example output from the CM model operating in a batch-culture mode. Here the prey are of low abundance. With increasing self-shading and the use of nitrate rather than ammonium after d10, ChlC and RuBisCO increase, falling on exhaustion of the DIN. Note that growth is simulated here in a 12hr:12h light:dark cycle, hence the oscillations or step-like changes in parameter values. Parameter abbreviations and units are given below.

Output parameters displayed in Fig.S2:

Name	Unit	Explanation
sysC	mgC m^{-3}	total system C
DOC	mgC m^{-3}	labile dissolved organic C
AA_C	mgC m^{-3}	dissolved amino acid C
DIC	mgC m^{-3}	dissolved inorganic C
C_prey1	mgC m^{-3}	prey#1 C
C_prey2	mgC m^{-3}	prey#2 C
VOC	mgC m^{-3}	voided organic C
ProtCTot	mgC m^{-3}	total protist C (i.e., ^{M}C + ^{C}C)
ChlC	g g^{-1}	chlorophyll : total protist C content
Rubpc	g g^{-1}	photosystem (chloroplast) : total protist C content
sysN	mgN m^{-3}	total system N
AA_N	mgN m^{-3}	dissolved amino acid N
NH4	mgN m^{-3}	ammonium-N
NO3	mgN m^{-3}	nitrate-N
N_prey1	mgN m^{-3}	prey#1 N
N_prey2	mgN m^{-3}	prey#2 N
VON	mgN m^{-3}	voided organic N
protN	mgN m^{-3}	total protist N
sysP	mgP m^{-3}	total system N
DIP	mgP m^{-3}	dissolved inorganic P
P_prey1	mgP m^{-3}	prey#1 P
P_prey2	mgP m^{-3}	prey#2 P
VOP	mgP m^{-3}	voided organic P
protP	mgP m^{-3}	total protist P
GCu	gC gC^{-1} d^{-1}	gross C-specific growth
Cfix	gC gC^{-1} d^{-1}	photosynthesis
BR	gC gC^{-1} d^{-1}	basal respiration
mavgCu	gC gC^{-1} d^{-1}	24hr moving average growth
UmRT	gC gC^{-1} d^{-1}	maximum growth rate at reference temperature
UmT	gC gC^{-1} d^{-1}	maximum growth rate at current temperature
Cfix	gC gC^{-1} d^{-1}	photosynthesis
mavgnetPS	gC gC^{-1} d^{-1}	24hr moving average photosynthesis
NCu	DL	N-status (1 being optimal)
PCu	DL	P-status (1 being optimal)
SCu	DL	diatom Si-status (1 being optimal)
NPSiCu	DL	N,P,Si,C-status (1 being optimal)
relU	DL	relative growth rate (1 being = UmT)
CCu	DL	C-status (1 being optimal)
cells	cell L^{-1}	cell abundance

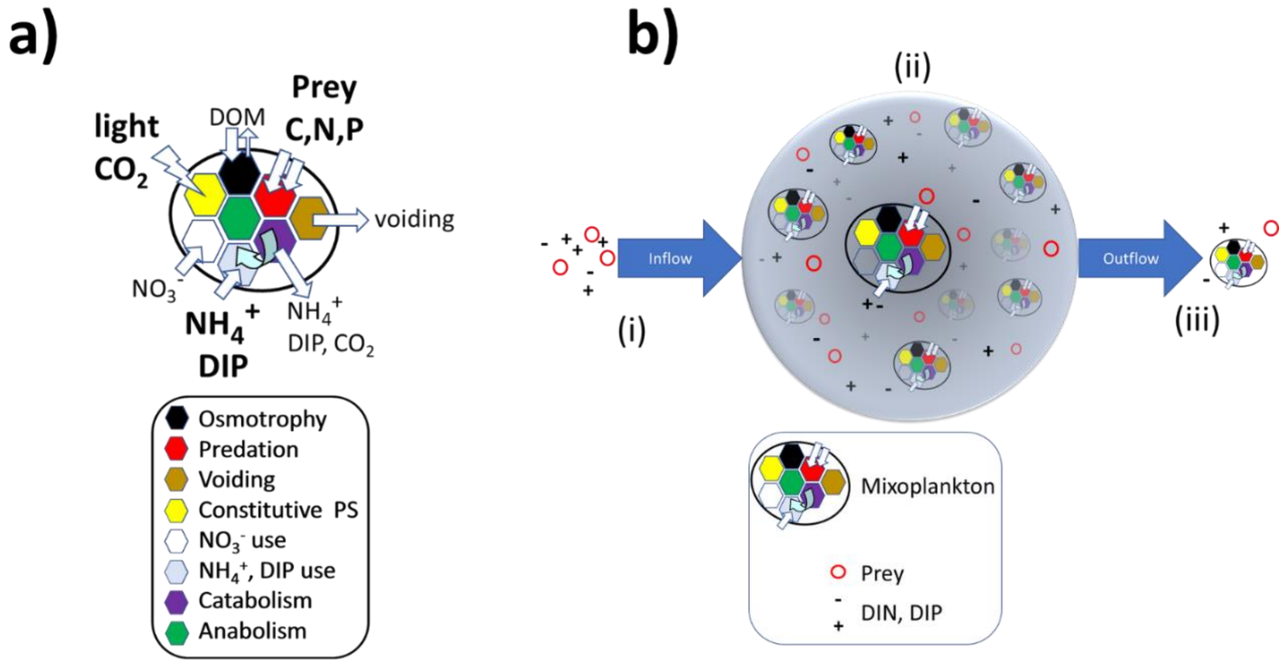

Fig. S3. Schematics of the constitutive mixoplankton (CM) model and the chemostat-style setup used for the analysis of synergy between phototrophy and phagotrophy. Panel (**a**) shows the physiological interconnectivity between different modes of nutrition in the mixoplankton model; those parts in bold were the focus of this work, with no supply of nitrate (NO_3^-), nor of dissolved organic matter (DOM). Mixoplankton growth was supported by carbon (C), nitrogen (N), and phosphorus (P) from phototrophy (photosynthesis + NH_4^+ + DIP) and phagotrophy (prey C,N,P). Note the internal cycling of NH_4^+ and DIP (dissolved inorganic phosphorus) through catabolism which negates specific dynamic action (SDA). See also Supplementary Information S1. Panel (**b**) shows the chemostat setup with: (i) inflows of resources as inorganic nutrients and prey; (ii) growth of the mixoplankton exploiting these resources; (iii) outflow of residual resources and mixoplankton, and also of voided and regenerated wastes (not shown). Inflow and outflow dilution rates were the same, and at steady state this dilution rate sets (is equal to) the growth rate averaged over the 24 h light-dark cycle.

Table S1. Prey allometry and stoichiometry. The carbon (C) per cell values were calculated from the general equation: pg C = a·(prey cell volume) ^b, where prey cell volume = 4/3 π(ESD/2)^3; values of a and b are indicated in the table; ESD: equivalent spherical diameter. i, Romanova & Sahzin, 2010; ii, Heywood et al., 2006; iii, Zimmermann et al., 2014; iv, Bertilsson et al., 2003; v, Tortell et al., 1996. From this, and the stoichiometric mass ratios are calculated the elemental content per cell; examples of calculated values for C, N and P are provided for cells of ESD 1 µm.

	**Bacteria**	**Prokaryote phytoplankton**
a	0.13[i]	0.22[ii]
b	0.44[i]	1[ii]
N:C (mass)	0.2427[iii]	0.2316[iv]
P:C (mass)	0.034[iii]	0.0165[iv]
fg C $cell^{-1}$ @ ESD 1 µm	97.79	115.19
fg N $cell^{-1}$ @ ESD 1 µm	23.73	26.67
fg P $cell^{-1}$ @ ESD 1 µm	0.81	0.44

Table S2. Concentrations of prey (as C, N, P) and inorganic nutrients (N and P) under different resource configurations. Values for cyanobacteria as prey are only given for the equal split (i.e., 50:50) between prey P and DIP. Total resource N was always 70 mgN m-3 (≡5μM if supplied 100% as DIN). The default proportion of system P as prey was 0.2 (lines in bold). The stoichiometry of cyanobacteria do not permit proportions of system P as cyanobacterial prey-P above 0.5.

			prokaryote prey load			inorganic nutrient load			
Regime N:P	Prey	proportion of P as prey	ugP/L	ugN/L	ugC/L	DIN (ugN/L)	DIP (ugP/L)	DIN:DIP	mole DIN:DIP
16	Bacteria	0.00	0.00	0.00	0.00	70.00	9.69	7.23	16.00
		**0.20**	**1.94**	**13.83**	**56.99**	**56.17**	**7.75**	**7.25**	**16.05**
		0.40	3.88	27.66	113.97	42.34	5.81	7.28	16.13
		0.50	4.84	34.58	142.46	35.42	4.84	7.31	16.19
		0.60	5.81	41.49	170.96	28.51	3.88	7.36	16.29
		0.80	7.75	55.32	227.94	14.68	1.94	7.58	16.78
		0.90	8.72	62.24	256.43	7.76	0.97	8.01	17.75
		1.00	9.69	69.15	284.93	0.85	0.00	-	-
32	Bacteria	0.00	0.00	0.00	0.00	70.00	4.84	14.45	32.00
		**0.20**	**0.97**	**6.92**	**28.49**	**63.08**	**3.88**	**16.28**	**36.05**
		0.40	1.94	13.83	56.99	56.17	2.91	19.33	42.80
		0.50	2.42	17.29	71.23	52.71	2.42	21.76	48.19
		0.60	2.91	20.75	85.48	49.25	1.94	25.42	56.29
		0.80	3.88	27.66	113.97	42.34	0.97	43.71	96.78
		0.90	4.36	31.12	128.22	38.88	0.48	80.27	177.75
		1.00	4.84	34.58	142.46	35.42	0.00	-	-
16	Cyanobacteria	0.00	0.00	0.00	0.00	70.00	9.69	7.23	16.00
		**0.20**	**1.94**	**27.20**	**117.42**	**42.80**	**7.75**	**5.52**	**12.23**
		0.40	3.88	54.39	234.85	15.61	5.81	2.69	5.95
		0.50	4.84	67.99	293.56	2.01	4.84	0.42	0.92
32	Cyanobacteria	0.00	0.00	0.00	0.00	70.00	4.84	14.45	32.00
		**0.20**	**0.97**	**13.60**	**58.71**	**56.40**	**3.88**	**14.56**	**32.23**
		0.40	1.94	27.20	117.42	42.80	2.91	14.73	32.61
		0.50	2.42	33.99	146.78	36.01	2.42	14.87	32.92
		0.60	2.91	40.79	176.14	29.21	1.94	15.07	33.38
		0.80	3.88	54.39	234.85	15.61	0.97	16.11	35.68
		0.90	4.36	61.19	264.20	8.81	0.48	18.19	40.28
		1.00	4.84	67.99	293.56	2.01	0.00	-	-

Supplementary Results

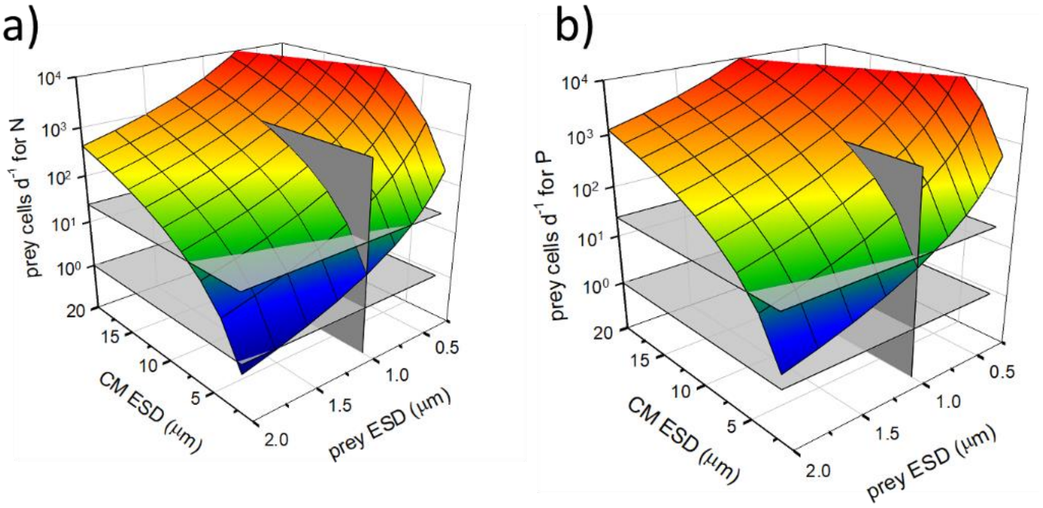

Fig. S4. Ingestion rate of prokaryotic cyanobacterial picophytoplankton prey by a constitutive mixoplankton (CM) predator of the indicated equivalent spherical diameters (ESD) required to support the structural demands of the CM at a growth rate of 1 cell division per day in terms of N-acquisition (panel a) or P-acquisition (panel b). See Fig. 2 legend for interpretation of these plots and Supplementary Information Table S1 for stoichiometric values.

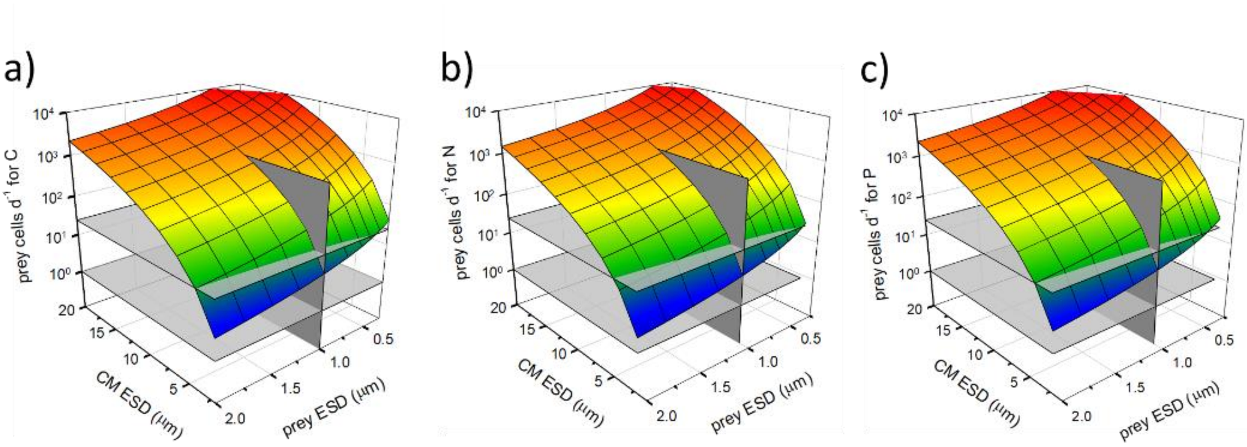

Fig. S5. Ingestion rate of bacteria prey by a constitutive mixoplankton (CM) predator of the indicated equivalent spherical diameters (ESD) required to support structural demands of the CM at a growth rate of 1 cell division per day in terms of C-acquisition (panel a), N-acquisition (panel b) or P-acquisition (panel c). See Fig. 2 legend for interpretation of these plots and Supplementary Information Table S1 for stoichiometric values.

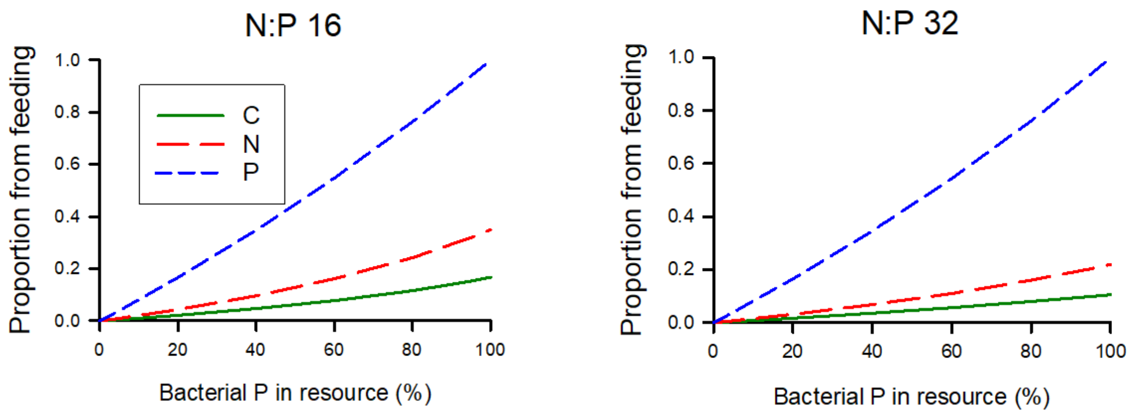

Fig. S6. Proportions of C, N and P acquired from feeding from the simulations shown in Fig. 4. See legend for Fig. 4 for details.

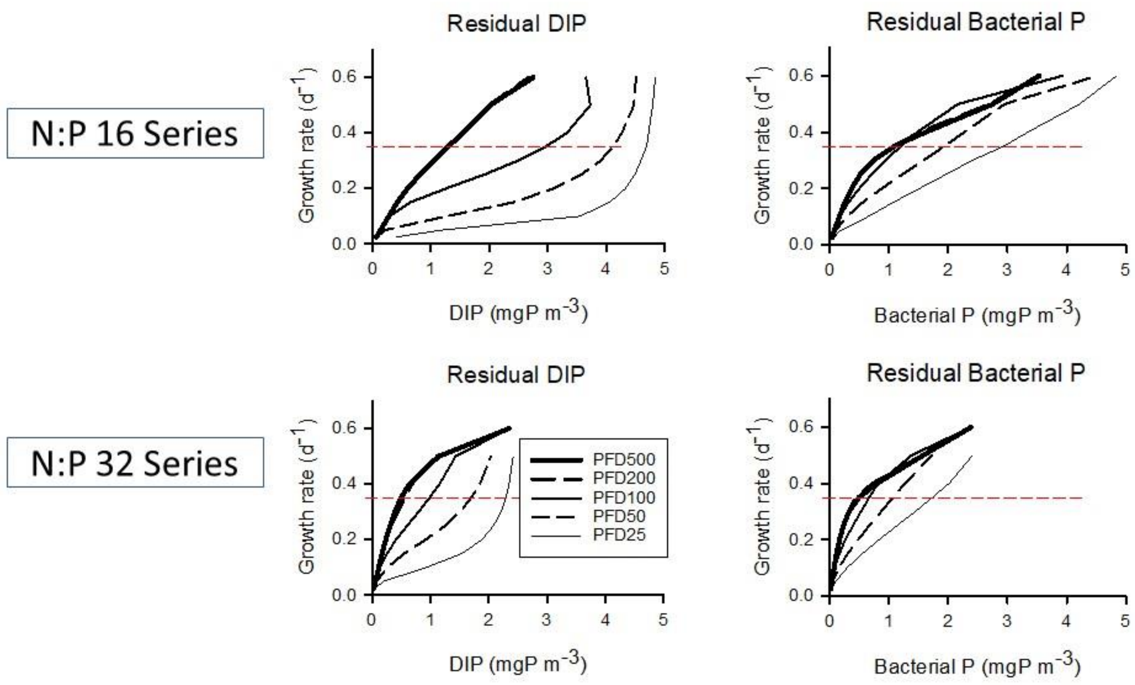

Fig. S7. Residual DIP and bacterial-P abundances for the simulations shown in Fig. 5. The dashed red line is at half maximum growth rate, indicating the values of $K_{0.5}^{DIN}$ and $K_{0.5}^{prey\text{-}P}$ by reading off the x-axis. The greater exploitation of prey-C at lower light requires higher ingestion rates which in turn raises the values of $K_{0.5}$.

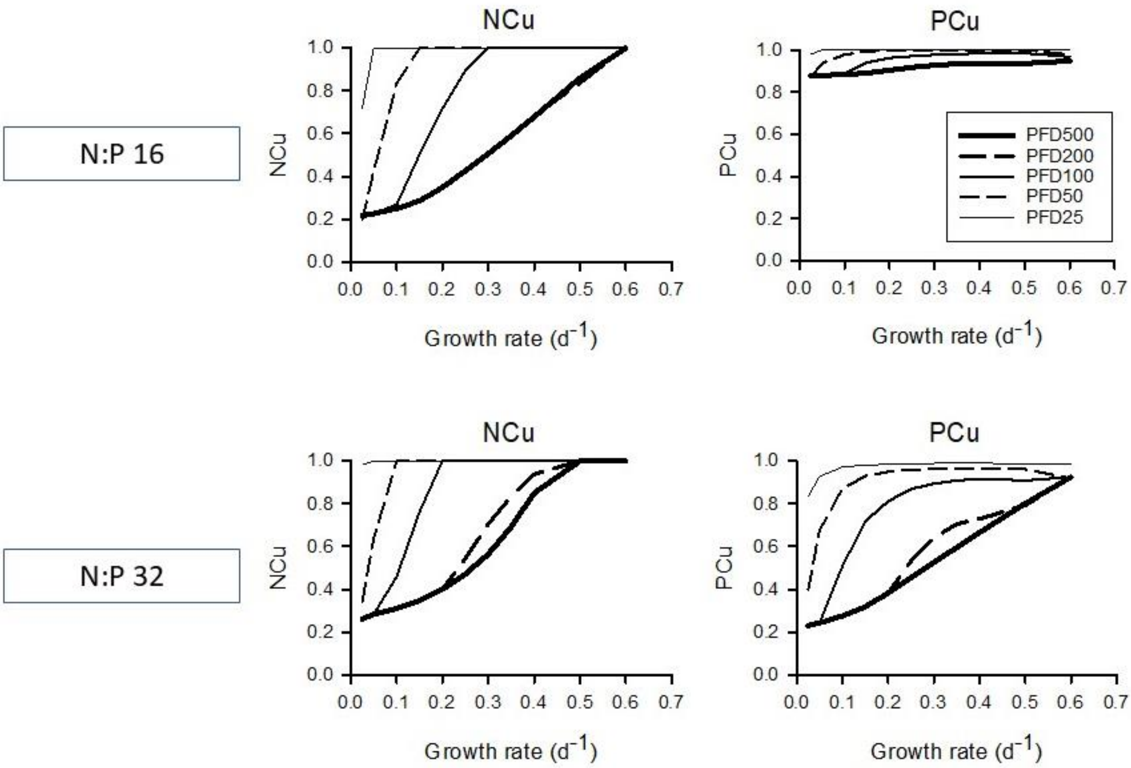

Fig. S8. Nitrogen (NCu) and Phosphorous (Pcu) nutrient status for the CM in the simulations shown in Fig. 5. Nutrient status ranges from 0 for full limitation (nutrient quota = minimum) to 1 (nutrient quota >= optimal).

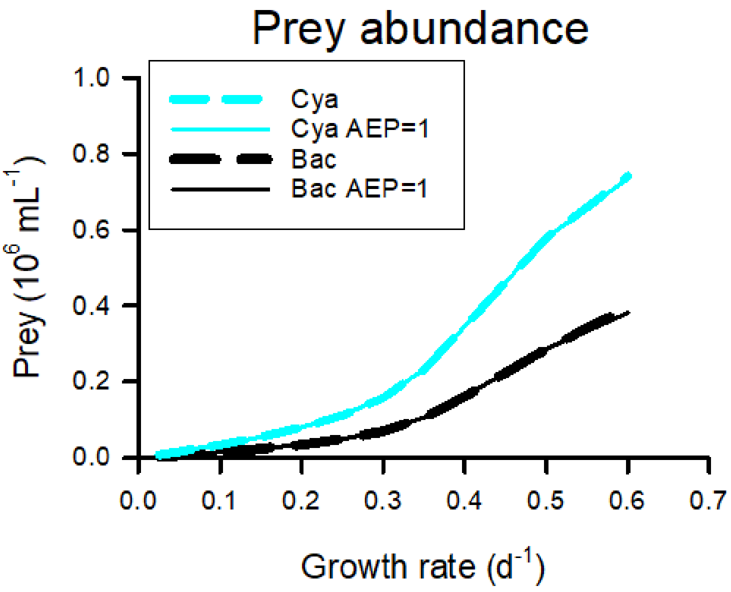

Fig. S9. Residual prey abundance in the systems portrayed in Fig. 6. All other details provided in the legend to Fig. 6.

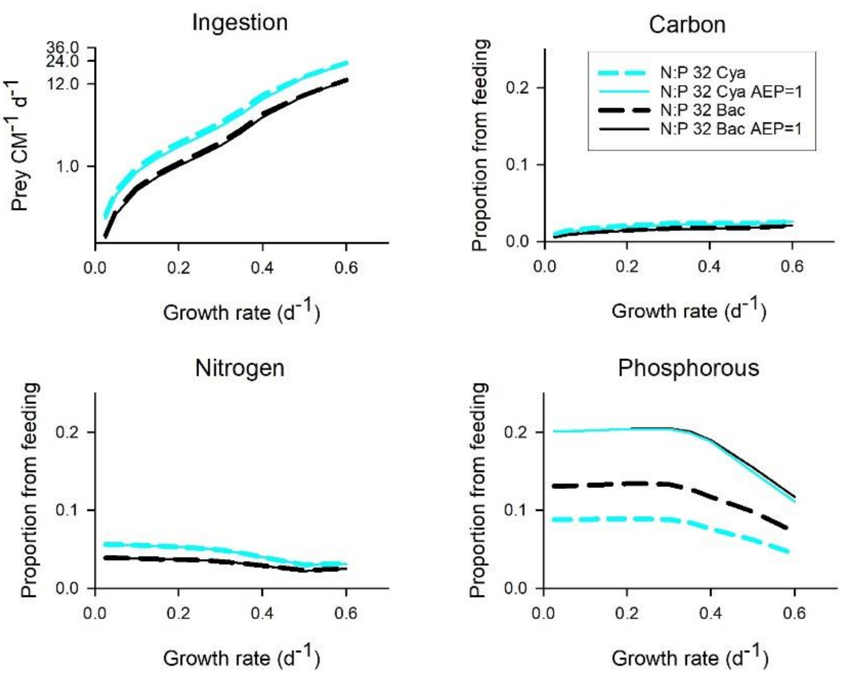

Fig. S10. Ingestion rates and fate of ingested prokaryote C,N,P biomass, with a resource regime of N:P=32. As for Fig. 6 (which is with N:P=16). See legend for Fig. 6 for all other details. This resource regime drives P-limitation of growth in the mixoplankton rather than N-limitation as in Fig. 6. In comparison with Fig. 6, the contributions from feeding to carbon and nitrogen are lower.

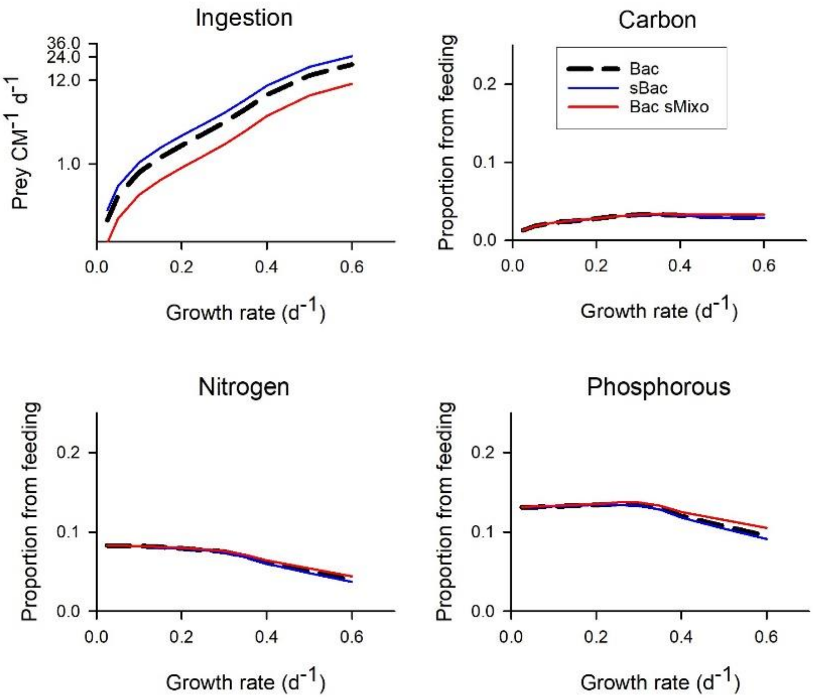

Fig. S11 Ingestion rates and fate of bacteria C,N,P biomass when CM predator or prokaryotic prey are smaller. As for Fig. 6, with 'Bac' the same as in that figure. Also shown here the situation with smaller bacteria (halved biomass; 0.8µm ESD rather than 1µm; 'sBac') and smaller CM (halved biomass, equating to ca. 4µm ESD rather than 5µm; 'sMixo'). See Fig. S12 for residual prey abundance.

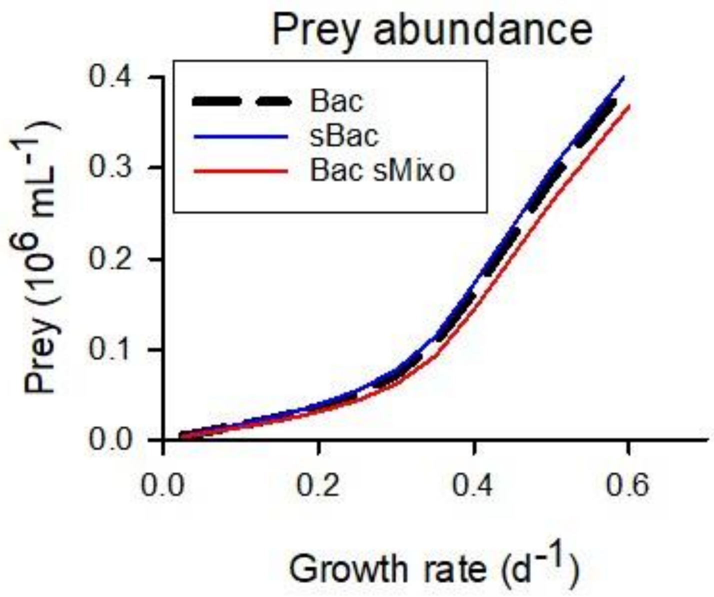

Fig. S12 Residual prey abundance in the systems portrayed in Fig. S11. All other details provided in the legend to Fig. S11.

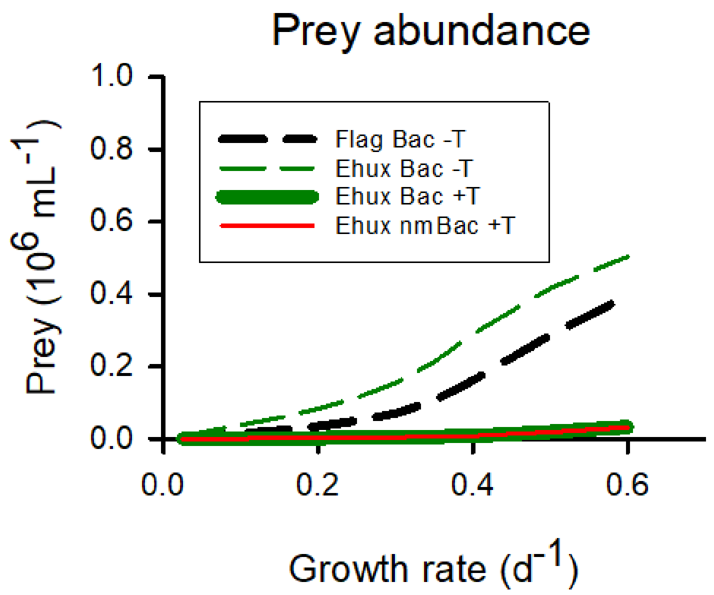

Fig. S13. Residual prey abundance in the systems portrayed in Fig. 7. All other details provided in the legend to Fig. 7.

Supplementary Discussion

Table S3. Examples of ingestion rates of bacteria into constitutive mixoplankton. Rates in the literature have been converted to common units of prey cells (predator cell)$^{-1}$ d^{-1}. Sizes of the mixoplankton where not available from the references listed in the table, have been obtained from the Mixoplankton Database (Mitra et al., 2023). *Fluorescent Labelled Bacteria. The 'community' feeding rates reflect average feeding across the community of cells in the culture, recognising that statistically only a few cells are seen to be feeding. The 'feeding cells' is the ingestion rate amongst cells that are all actively feeding.

Species	Size (µm)	Ingestion rate (d^{-1})	References
Calcidiscus leptoporus	11 × 11	2.16*	Avrahami and Frada, 2020
Calyptrosphaera sphaeroidea	8 × 8	0.72*	Avrahami and Frada, 2020
Haptolina ericina	6.5 × 6.5	192-432	Nygaard and Tobiesen, 1993
Isochrysis galbana:	5-6 × 2-4		Anderson et al., 2018
community		24	
feeding cells		276	
Nephroselmis pyriformis:	4-7.5 × 5-6		Anderson et al, 2018
community		4.8-21.6	
feeding cells		28.8-45.6	
Ochromonas sp.	12 × 20	101.45-289.86	Wilken et al., 2020
Phaeocystis globosa	3 × 10.4	0.288-1.92	Koppelle et al., 2022
Prymnesium parvum	10.5 × 5.8	28.8-139.2	Nygaard and Tobiesen, 1993; Legrand, 2001
Teleaulax amphioxeia	11.5 × 5.8	6.24-17.76	Yoo et al., 2017

References

Anderson, R, Jürgens, K, Hansen, PJ. Mixotrophic phytoflagellate bacterivory field measurements strongly biased by standard approaches: a case study. Front. Microbiol. 2017; 8:1398.

Avrahami, Y, Frada, MJ. Detection of phagotrophy in the marine phytoplankton group of the coccolithophores (Calcihaptophycidae, Haptophyta) during nutrient - replete and phosphate - limited growth. J. Phycol. 2020; 56:1103-1108.

Bertilsson, S, Berglund, O, Karl, DM, Chisholm, SW. Elemental composition of marine *Prochlorococcus* and *Synechococcus*: Implications for the ecological stoichiometry of the sea. Limnol. Oceanogr. 2003; 48:1721-1731.

Cunningham, BR, John, SG. The effect of iron limitation on cyanobacteria major nutrient and trace element stoichiometry. Limnol. Oceanogr. 2017; 62:846-858.

Fasham, MJR, Ducklow, HW, Mckelvie, SM. A nitrogen-based model of plankton dynamics in the oceanic mixed layer. J. Mar. Res. 1990; 48:591-639.

Flynn, KJ, Mitra, A. Building the "perfect beast": modelling mixotrophic plankton. J. Plankton Res. 2009: 31:965-992.

Flynn, KJ, Mitra, A. Why plankton modelers should reconsider using rectangular hyperbolic (Michaelis-Menten, Monod) descriptions of predator-prey interactions. Front. Mar. Sci. 2016; 3:165.

Flynn, KJ, Mitra, A. DRAMA - a cybernetic approach for Plankton Digital Twins (Version v1) Zenodo 2023. https://doi.org/10.5281/zenodo.7848329

Flynn, KJ, Torres, R, Irigoien, X, Blackford, JC. Plankton digital twins—a new research tool. J. Plankton Res. 2022; 44:805-813.

Geider, R.J., LaRoche, J., 2002. Redfield revisited: variability of C:N:P in marine microalgae and its biochemical basis. Eur. J. Phycol. 37, 1-17.

Heldal, M, Fagerbakke, KM, Tuomi, P, Bratbak, G. Abundant populations of iron and manganese sequestering bacteria in coastal water. Aquat. Microb. Ecol. 1996; 11:127-133.

Heywood, JL, Zubkov, MV, Tarran, GA, Fuchs, BM, Holligan, PM. Prokaryoplankton standing stocks in oligotrophic gyre and equatorial provinces of the Atlantic Ocean: evaluation of inter-annual variability. Deep Sea Res. II 2006; 53:1530-1547.

Johansen, JE, Pinhassi, J, Blackburn, N, Zweifel, UL, Hagström Å. Variability in motility characteristics among marine bacteria. Aquat. Microb. Ecol. 2002; 28:229-237.

Koppelle, S, López-Escardó, D, Brussaard, CP, Huisman, J, Philippart, CJ, Massana, R, et al. Mixotrophy in the bloom-forming genus *Phaeocystis* and other haptophytes. Harmful Algae 2022; 117:102292.

Legrand, C., 2001. Phagotrophy and toxicity variation in the mixotrophic *Prymnesium patelliferum* (Haptophyceae). Limnology and Oceanography 46, 1208-1214.

Leles, SG, Polimene, L, Bruggeman, J, Blackford, J, Ciavatta, S, Mitra, A, et al. Modelling mixotrophic functional diversity and implications for ecosystem function. J. Plankton Res. 2018; 40:627-642.

Leles, SG, Mitra, A, Flynn, KJ, Tillmann, U, Stoecker, D, Jeong, HJ, et al. Sampling bias misrepresents the biogeographic significance of constitutive mixotrophs across global oceans. Glob. Ecol. Biogeogr. 2019; 28:418-428.

Leles, SG, Bruggeman, J, Polimene, L, Blackford, J, Flynn, KJ, Mitra, A. Differences in physiology explain succession of mixoplankton functional types and affect carbon fluxes in temperate seas. Prog. Oceanogr. 2021; 190:102481.

Li, WK. Annual average abundance of heterotrophic bacteria and *Synechococcus* in surface ocean waters. Limnol. Oceanogr. 1998; 43:1746-1753.

Lin, C-H, Flynn, KJ, Mitra, A, Glibert, PM. Simulating effects of variable stoichiometry and temperature on mixotrophy in the harmful dinoflagellate *Karlodinium veneficum*. Front. Mar. Sci. 2018; 5:320.

Menden-Deuer, S, Lessard, EJ. Carbon to volume relationships for dinoflagellates, diatoms, and other protist plankton. Limnol. Oceanogr. 2000; 45:569-579.

Mitra, A, Flynn, KJ, Burkholder, JM, Berge, T, Calbet, A, Raven, JA, et al. The role of mixotrophic protists in the biological carbon pump. Biogeosci. 2014; 11:995-1005.

Mitra, A, Flynn, KJ, Tillmann, U, Raven, JA, Caron, D, Stoecker, DK, et al. Defining planktonic protist functional groups on mechanisms for energy and nutrient acquisition: incorporation of diverse mixotrophic strategies. Protist 2016; 167:106-120.

Mitra, A, Flynn, KJ, Anestis, K, Mansour, JS, Ferreira, GD, Calbet A. Novel Approaches For Investigating Marine Planktonic Mixotrophy. Zenodo. 2021 https://doi.org/10.5281/zenodo.5148500

Mitra, A, Caron, DA, Faure, E, Flynn, KJ. Gonçalves Leles, S, Hansen, PJ, et al. The mixoplankton database: Diversity of photo-phago-trophic plankton in form, function, and distribution across the global ocean. J. Eukaryot. Microbiol.; e12972. https://doi.org/10.1111/jeu.12972

Nelson, DM, Brand, LE. Cell division periodicity in 13 species of marine phytoplankton on a light:dark cycle. J. Phycol. 1979; 15:67–75.

Nygaard, K, Tobiesen, A. Bacterivory in algae: A survival strategy during nutrient limitation. Limnol. Oceanogr. 1993; 38:273–279.

Romanova, ND, Sazhin, AF. Relationships between the cell volume and the carbon content of bacteria. Oceanology 2010; 50:522-530.

Rothschild, BJ, Osborn, TR. Small-scale turbulence and plankton contact rates. J. Plankton Res. 1998; 10:465-474.

Tortell, PD, Maldonado, MT, Price, NM. The role of heterotrophic bacteria in iron-limited ocean ecosystems. Nature 1996; 383:330-332.

Unrein, F, Gasol, JM, Not, F, Forn, I, Massana, R. Mixotrophic haptophytes are key bacterial grazers in oligotrophic coastal waters. ISME J. 2014; 8:164-176.

Wilken, S, Choi, CJ, Worden, AZ. Contrasting mixotrophic lifestyles reveal different ecological niches in two closely related marine protists. J. Phycol. 2020; 56:52-67.

Yoo, YD, Seong, KA, Jeong, HJ, Yih, W, Rho, JR, Nam, SW, et al. Mixotrophy in the marine red-tide cryptophyte *Teleaulax amphioxeia* and ingestion and grazing impact of cryptophytes on natural populations of bacteria in Korean coastal waters. Harmful Algae 2017; 68:105-117.

Zimmerman, AE, Allison, SD, Martiny, AC. Phylogenetic constraints on elemental stoichiometry and resource allocation in heterotrophic marine bacteria. Env. Microbiol. 2014; 16:1398-1410.

Zubkov, MV, Tarran, GA. High bacterivory by the smallest phytoplankton in the North Atlantic Ocean. Nature 2008; 455:224-226.
